# Pliable, Scalable, and Degradable Scaffolds with Varying Spatial Stiffness and Tunable Compressive Modulus Produced by Adopting a Modular Design Strategy at the Macrolevel

**DOI:** 10.1021/acspolymersau.1c00013

**Published:** 2021-08-12

**Authors:** Hailong Liu, Shubham Jain, Astrid Ahlinder, Tiziana Fuoco, T. Christian Gasser, Anna Finne-Wistrand

**Affiliations:** † Department of Fibre and Polymer Technology, KTH Royal Institute of Technology, 100 44, Stockholm, Sweden; ‡ Solid Mechanics, Department of Engineering Mechanics, KTH Royal Institute of Technology, 100 44, Stockholm, Sweden; § Faculty of Health Sciences, University of Southern Denmark, 5230, Odense, Denmark; ⊥ Department of Engineering Mechanics, KTH Royal Institute of Technology, 100 44, Stockholm, Sweden

**Keywords:** degradable polymer, 3D printing, modular scaffold design, finite element analysis, adipose tissue regeneration, breast reconstruction

## Abstract

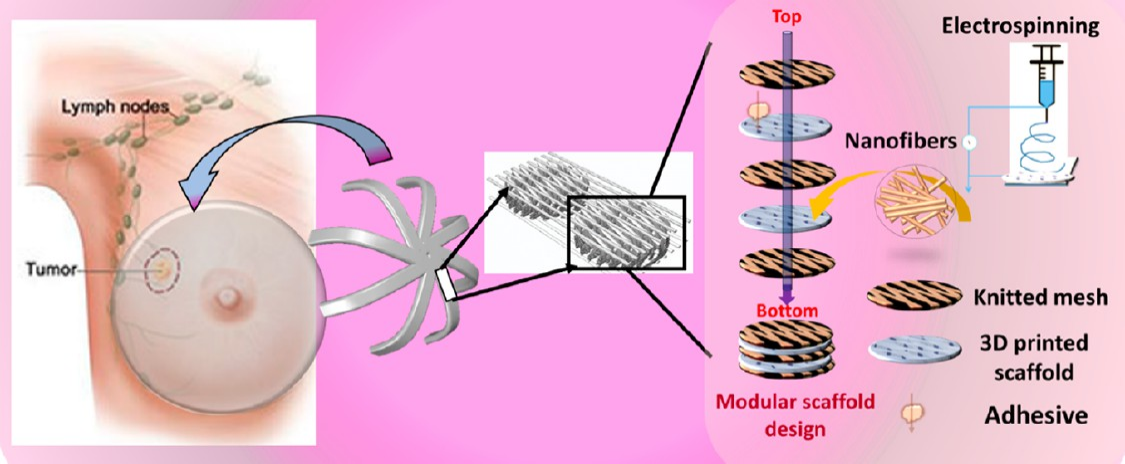

Clinical results obtained when degradable polymer-based medical devices are used in breast reconstruction following mastectomy are promising. However, it remains challenging to develop a large scaffold structure capable of providing both sufficient external mechanical support and an internal cell-like environment to support breast tissue regeneration. We propose an internal-bra-like prototype to solve both challenges. The design combines a 3D-printed scaffold with knitted meshes and electrospun nanofibers and has properties suitable for both breast tissue regeneration and support of a silicone implant. Finite element analysis (FEA) was used to predict the macroscopic and microscopic stiffnesses of the proposed structure. The simulations show that introduction of the mesh leads to a macroscopic scaffold stiffness similar to the stiffness of breast tissue, and mechanical testing confirms that the introduction of more layers of mesh in the modular design results in a lower elastic modulus. The compressive modulus of the scaffold can be tailored within a range from hundreds of kPa to tens of kPa. Biaxial tensile testing reveals stiffening with increasing strain and indicates that rapid strain-induced softening occurs only within the first loading cycle. In addition, the microscopic local stiffness obtained from FEA simulations indicates that cells experience significant heterogeneous mechanical stimuli at different places in the scaffold and that the local mechanical stimulus generated by the strand surface is controlled by the elastic modulus of the polymer, rather than by the scaffold architecture. From *in vitro* experiments, it was observed that the addition of knitted mesh and an electrospun nanofiber layer to the scaffold significantly increased cell seeding efficiency, cell attachment, and proliferation compared to the 3D-printed scaffold alone. In summary, our results suggest that the proposed design strategy is promising for soft tissue engineering of scaffolds to assist breast reconstruction and regeneration.

## 1 Introduction

Breast cancer is one of the commonest cancer forms globally, with approximately 2.2 million new cases in 2020.^1^ Typical treatment involves partial or complete surgical removal of the breast followed by reconstructive surgery.^2,3^ Reconstructive surgery often includes the insertion of silicone implants, autologous tissue flaps, or injection of adipose tissue. A suboptimal outcome is, however, not uncommon. Tissue flaps can result in recipient site morbidity due to lack of vascularization and injection of adipose tissue can result in volume loss during the initial 2–3 months due the lack of a mechanical support, both requiring repeated surgeries.^4−6^ In the case of silicone breast implants, capsular contracture of the surrounding tissue and migration of the implants resulting in ptosis of the breast are some of the negative issues encountered.^3,7^ Fibrosis has been shown to be mitigated by decreasing the stiffness of the implant or by the inclusion of growth factors that inactivate mechanical activation of myofibroblasts.^8^ Here we seek to demonstrate the development of a scalable device using a modular scaffold designed with finite element analysis (FEA) to enhance the amount of tissue around a breast implant. The architecture of the modular design internal bra was realized by combining a commercial degradable mesh with advanced additive manufacturing (AM) techniques on both the macroscale and nanoscale level through fused filament fabrication (FFF) and electrospinning.

**Figure 1:**
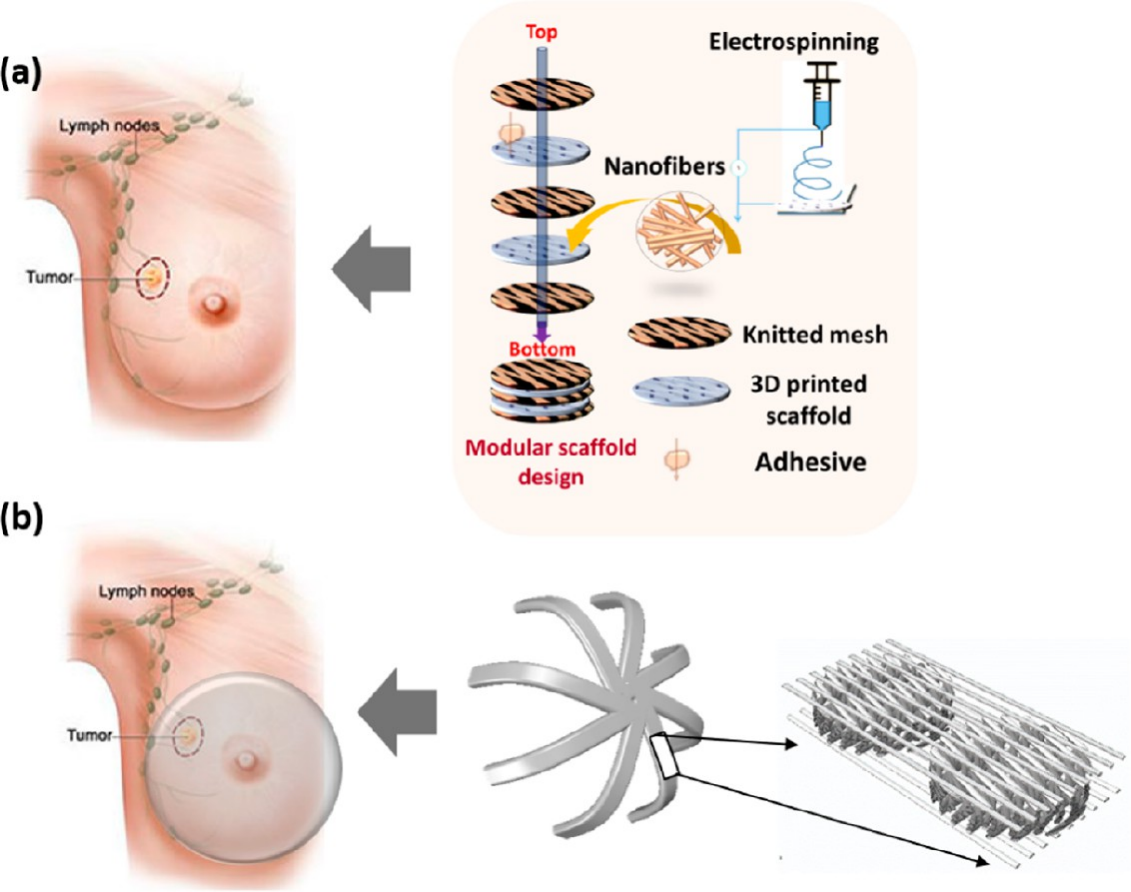
Design architecture of the modular scaffold designs forming an internal bra-like structure in breast reconstruction (a) for the purpose of lumpectomy showing the 3D printed design, the 3D printed structure with electrospun fibers, and the 3D printed structure with a mesh (b) showing how printed scaffold units can be combined using a textile mesh to form a larger implant.

The use of a mesh to add scaffold units together permits formation of a small device for lumpectomy reconstruction where only a small amount of tissue has been removed, a larger device to be used in conjunction with a silicone breast implant, or a larger strap-like device up to a bra-like prototype to aid in breast construction (Figure 1). Furthermore, a patient-specific prototype can be made by adjusting the length, number, and orientation of modular scaffold units to match the defect, size, and shape of the breast.

Poly(ε-caprolactone-co*-p*-dioxanone) (PCLDX) has previously been developed within the research group for adipose tissue regeneration.^9,10^ This polymer was herein 3D-printed into a gradient based scaffold design (G15) using FFF previously developed to adapt mechanical properties to that of breast tissue to shield the fragile adipocytes from external deformation.^11^ It has successfully been shown that a tissue chamber or a scaffold can be utilized to prevent tissue resorption by shielding the fragile adipose tissue from external deformation using polymers with a long degradation time such as poly(ε-caprolactone) (PCL), or nondegradable acrylic chambers.^12−17^

One of the challenges in large tissue constructs is to achieve vascularization, and another is for the new tissue to have enough space to expand at an adequate rate. Large pores and material degradation are therefore important features for a successful tissue regeneration. This must be tailored both through the polymer synthesis adapting the degradation time and the design of the implant to enhance pliability and large pore structures while providing a large surface area for cell attachment. Although 3D- printing allows for precise geometries to be manufactured that can shield the cells and provide the potential for personalized implants, the resolution of FFF technology does not give rise to a large surface area as a microenvironment for the cells, this can be made through electrospinning.^18,19^ Electrospun nanofibers facilitate cell adhesion, proliferation, and differentiation due to their high surface-to-volume ratio. They can mimic extracellular matrix (ECM) and contain compounds which could aid in the regeneration process.^20−22^ The use of hybrid scaffolds, also called biphasic scaffolds, showing combined approaches between the AM realm and 3D-printing with electrospinning, porogens, gels, or textiles are increasingly being reported.^23−25^

The 3D printed structures were herein combined with a commercially available degradable mesh, TIGR Matrix Surgical Mesh(TIGR Matrix). It has been shown that a synthetic degradable mesh can be used to keep the implant in place as an internal bra and reduce the risk of capsular contracture.^26,27^ The meshes provide pliability and tensile strength to keep the breast implant in place but do not aid in creating tissue volume. To understand how the addition of mesh influences the modular scaffold unit’s mechanical behaviors at both the macro- (scaffold unit stiffness) and microlevels (local mechanical stimulus within the scaffold), we used FEA simulations. FEA is a useful tool that is often used in tandem with AM to screen designs before fabrication.^6^ We have previously validated both the simulation of the 3D-printed scaffolds with experimental testing and how design can reduce mechanical stiffness to suit adipose tissue.^11^

We hypothesize that to have a role as an internal bra, a scaffold should not match the adipose tissue’s mechanical properties in the early stages of regeneration but rather those of the fibrous fascia that shield the cells from external deformations. Aside from adipose tissue, breast tissue is heterogeneous and consists of glands and a fibrous fascia known as Cooper’s ligament, or suspensory ligaments penetrating the tissue.^28−30^ The fibrous fascia and collagen fibers are unevenly distributed, and their distribution varies depending on the patient, age, and from where the sample is isolated. This heterogeneous structure is the reason why mechanical properties of breast tissue differ significantly from those of pure adipose tissue and renders their assessment difficult.^31−33^ Over time, scaffold degradation and the consequent decrease in mechanical properties will stimulate native tissue growth and scaffold replacement. This requires a pliable scaffold with a more rapid degradation time then that for PCL, others have suggested a need for a supportive structure for 6–8 months or up to a year.^12,14^ This correlates with our initial degradation data provided on thin PCLDX films, which showed a bulk degradation with polymer reaching the threshold for intracellular digestion in a time frame of 9 months.^10,34^ It also correlates with the degradation of the mesh. The TIGR Matrix consists of two aliphatic polyesters fibers, one where the mechanical properties are retained for 2 weeks, resulting in a reduced stiffness after this time frame.^35^ The slower degrading fiber has been found to be macroscopically undetectable 36 months after implantation in sheep, but its mechanical properties start to decrease after 6 months.

Herein, we engineered pliable modular scaffolds that can be used as internal bras to support adipose tissue regeneration. The modular scaffold was designed to be scalable while maintaining its pliability, consisting of the 3D-printed G15 structure to create volume and to shield the cells from external deformation. The modular scaffold has mechanical properties over different length scales. This makes it possible to adapt local stiffness for the cells, and the overall scaffold stiffness can withstand external forces. The assembly process for these designs has been developed, and their geometry and mechanical properties have been characterized using scanning electron microscopy (SEM) and mechanical testing. Human dermal primary fibroblast (HDFs) cells were used for a preliminary study on initial cell attachment and proliferation within the modular scaffold.

## 2 Materials and Methods

### 2.1 Synthesis of poly(ε-caprolactone-*co*-*p*-dioxanone)

Poly(ε-caprolactone-*co*-*p*-dioxanone), PCLDX, was synthesized by ring-opening copolymerization in bulk ε-caprolactone (CL) and *p*-dioxanone (DX), following a previously developed procedure for large-scale synthesis.^10^ Briefly, DX (77 g; 0.75 mol) and CL (473 g; 4.14 mol) were mixed in a one-liter round-bottom flask at 50 °C; ethylene glycol (0.185 g; 3.00 mmol) and tin octanoate (0.150 g; 0.37 mmol), which act as an initiator and catalyst, respectively, were added to the monomer mixture and stirred for 3 min. The polymerization mixture was transferred to a stainless-steel reactor covered with a Teflon film (Chemistik 100–3 s, Chemistik Systems, Poland). The reactor was then sealed and transferred to an oven set at 130 °C, where the copolymerization proceeded over 64 h. The as-polymerized PCLDX was cooled in liquid nitrogen and milled into granules of approximately 3 mm diameter using an IKA MF 10 basic milling head (IKA-Werke, Germany).

### 2.2 Preparation of the Adhesive

The adhesive consisted of a 20 wt % solution of poly(d,l-lactide-cotrimethylene carbonate), PDLTMC, in ethyl acetate and was prepared as previously reported.^36^ Briefly, PDLTMC with a trimethylene carbonate (TMC) content of 11 mol % was synthesized by ring-opening copolymerization in bulk d,l-lactide (d,l-LA; 10.0 g; 0.069 mol) and TMC (0.787 g; 7.71 mmol) using tin octanoate as the catalyst (526 μL of 0.122 M solution in toluene; 64 μmol) and ethylene glycol as the initiator (542 μL of 0.474 M solution in toluene; 542 μmol). Copolymerization was performed at 130 °C for 48 h. To prepare the adhesive solution, 200 mg of as-polymerized copolymer was dissolved in 1 mL of ethyl acetate.

### 2.3 Filament Spinning

The filaments used for fused filament fabrication were melt-spun using a Felfil filament extruder (Collettivo Cocomeri, Italy). The extruder was equipped with a nozzle 3 mm in diameter, operated at 145 °C and used an in-house winder with a rotating drum 300 mm in diameter. It rotated at 9 rpm and resulted in filaments of approximately 1.75 mm in diameter.

### 2.4 Fused filament fabrication (FFF)

The PCLDX was printed into our previously developed G15 design for adipose tissue.^11^ The G15 design is formed by six layers of printed strands, each of which is rotated by 15 degrees. The strand distance in the six layers changes according to 0.8–0.8–0.4–0.4–0.8–0.8 mm, which results in a pore size gradient. Rectangular sheets of the G15 design with dimensions of 50 mm × 25 mm were manufactured using an FFF Original Prusa i3MK3S 3D printer (Prusa Research a.s, Czech Republic), using a nozzle temperature of 170 °C and nozzle diameter of 0.4 mm while keeping the bed at room temperature. During the printing process, the fan was set to 100%, and the extruder hose was cooled using icepacks that had been cooled in dry ice and acetone. The temperature of the extruder head was monitored with an infrared thermometer and stayed below 20 °C to allow for feeding to take place at a temperature near the crystallization temperature (*T*_c_) of the PCLDX. PrusaSlicer-2.1.0 (Prusa Research a.s, Czech Republic) was used to slice the STL file and convert it to gcode with the desired printing parameters. The gcode was further processed to adjust the printing path using Python 3.7.4 in Visual Studio code (Microsoft, USA) through Anaconda Navigator (Anaconda Inc., US.A).The printer head was cooled using dry ice during printing. The sheets were fabricated into three layers with strand distances of 0.8–0.8–0.4 mm and six layers with strand distances of 0.8–0.8–0.4–0.4–0.8–0.8 mm to allow for assembly of the modular scaffold with the TIGR Matrix (Novus Scientific, Sweden) and the degradable adhesive. Scaffolds of 8 mm or 5 mm were manually punched out of the sheets using a die-punch (Slöjdetaljer, Sweden).

### 2.5 Preparation of the Nanofibers

Nanofibers were prepared using an in-house-made electrospinning machine. After solvent optimization, a 75:25 solvent mixture of 2,2,2-trifluoroethanol (TFE) and chloroform was used to dissolve poly(l-lactide-*co*-trimethylene carbonate) (PLATMC, kindly provided by Novus Scientific AB, Sweden) (*M*_n_ ∼ 220 kg mol^–1^) (10% w/v). A 5 mL syringe was filled with the solution and then pumped (Harvard apparatus, USA) at a flow rate of 5 mL h^–1^ through a 22-gauge size at a voltage of 15 kV. The 3D-printed PCLDX scaffolds were placed on a plate collector, and nanofibers were collected onto the scaffolds for 5 min. The 3D printed PCLDX scaffolds with nanofibers were splutter coated with Pt:Pd using a Cressington 208HR High Resolution Sputter Coater with a Pt:Pd target before micrographs of the nanofibers were taken with a high field emission scanning electron microscope S-4800 (SEM) (Hitachi, Japan) and then processed with ImageJ (NIH, USA.).

### 2.6 Differential Scanning Calorimetry (DSC)

DSC using a DSC 1 instrument (Mettler Toledo, USA), calibrated with indium, was performed to measure the thermal properties in aluminum pans. Measurements were performed under nitrogen flow (50 mL min ^–1^) with a heating rate of 10 °C min ^–1^ from −20 to 120 °C.

### 2.7 Size Exclusion Chromatography (SEC)

The number-average molar mass (*M*_n_) and dispersity (*Đ*) of the polymers were measured by SEC at 35 °C on a Malvern GPCmax (Malvern Panalytical, UK) equipped with an autosampler, a PLgel 5 μm guard column (7.5 × 50 mm), two PLgel 5 μm MIXED-D (300 × 7.5 mm) columns and a Viscotek RI detector. CHCl_3_ was used as the eluent at a flow rate of 0.5 mL min^–1^. Polystyrene standards with narrow dispersity were used for calibration, and the flow rate fluctuations were corrected using toluene as an internal standard. Three measurements were performed for each sample, and the average value is reported.

### 2.8 Tabletop Scanning Electron Microscopy (SEM)

The top section of the G15 and G15 with cross section of the modular scaffold designs was visualized using a tabletop scanning electron microscope (SEM) TM-1000 (Hitachi, Japan) with an acceleration voltage of 15 kV after immersion of the samples into liquid nitrogen and sectioning. No conductive coating was used for tabletop SEM evaluation. Images were acquired at a magnification of 50×.

### 2.9 Construction of the Modular Scaffold Unit Design

To construct a modular unit scaffold design which can be extended into a thicker internal bra, Figure 1a, using the TIGR Matrix (kindly donated from Novus Scientific, Sweden), the PCLDX synthesized according to section 2.1 was spun into filaments according to section 2.2 and 3D-printed into either three-layered or six-layered sheets according to the G15 as outlined in section 2.3. Three combinations were explored (shown in Table 1); the TIGR Matrix was either glued in one side (OS), on both sides (TS), or on both sides and in the middle (TSM) of the G15 design, with synthetic degradable adhesive using a chopstick or a large needle with the top of the eye-section cut off as an applicator.

**Table 1:**
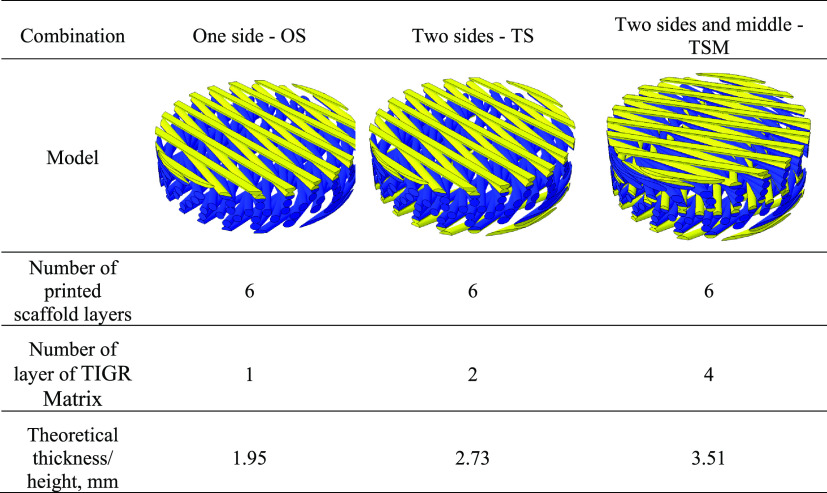
Outline of the Three Modular Scaffold Designs Where the Mesh Position Relative to the Scaffold Describes the Name^a^

a The yellow section is the TIGR Matrix and the blue section is the 3D-printed PCLDX scaffold.

For cell culture studies the TSM model was further enhanced by an electrospun nanofiber layer replacing the middle TIGR Matrix layer called TSM_NF.

### 2.10 Computational Analysis Using Finite Element Analysis

The mechanical properties of the OS, TS, and TSM designs were investigated with small-strain FEM models (ABAQUS 6.14 assembly module, Dassault Systemes, France). The 3D printed PCLDX was described as a homogeneous, isotropic, and linear elastic material with an elastic modulus of 210 MPa obtained from our previous tensile measurements of extruded filaments, while the Poisson’s ratio was simply set to 0.3.^9^ PCL was used as a control in the simulation, with the value of the elastic modulus also taken from previous tensile measurements. However, the complexity of the mesh structure makes it difficult to replicate in the simulation model. Instead, extra layers were added to the G15 scaffold, and the elastic modulus of the mesh was assigned. The diameter of the OS, TS, and TSM designs was set to 10 mm. The elastic modulus of the modular scaffolds designs was simulated in compression between two rigid plates. A displacement of modular scaffolds/devices equal to 1% of their thickness (corresponding to −1% strain) was applied on the top rigid plate, while the bottom rigid plate was fully constrained. Ten-node tetrahedral (C3D10) elements and a mesh size of approximately 0.2 mm represented the spatial discretization. A surface-to-surface contact model with a frictional coefficient of 0.2 prescribed the contact, and the effective compressive modulus was then calculated from the computed reaction force.^37^Table 2 gives an overview of the input values in the model.

**Table 2:** Input Values and Outline of the Compression Simulation

	Scaffold + TIGR Matrix		
Part type	Solid		Solid
Modular design	G15 design		Mesh
Pore size, mm	0.8–0.8–0.4–0.4–0.8–0.8		
	0.6–0.6–0.3–0.3–0.6–0.6		
	0.4–0.4–0.2–0.2–0.4–0.4		
Material	PCL	PCLDX	TIGR Matrix
Elastic modulus (MPa)	320	210	40
Boundary condition	Displacement equals to 1% strain applied on the rigid plate at top side of the scaffold		

#### 2.10.1 Simulation of a Device

To evaluate the feasibility of scale up for the OS, TS, and TSM designs to a device connected by an extended piece of TIGR Matrix, compression simulation was carried out on two scaffolds placed at different distances (*D*) of 0.8, 2, 4.4, and 8 mm. The width of the device was equal to the diameter of the modular scaffold unit, 10 mm, and the length of the device was the sum of the diameter of two modular scaffold units and the distance, 20+D mm. Further information regarding the simulation is given in Table 3.

**Table 3:**
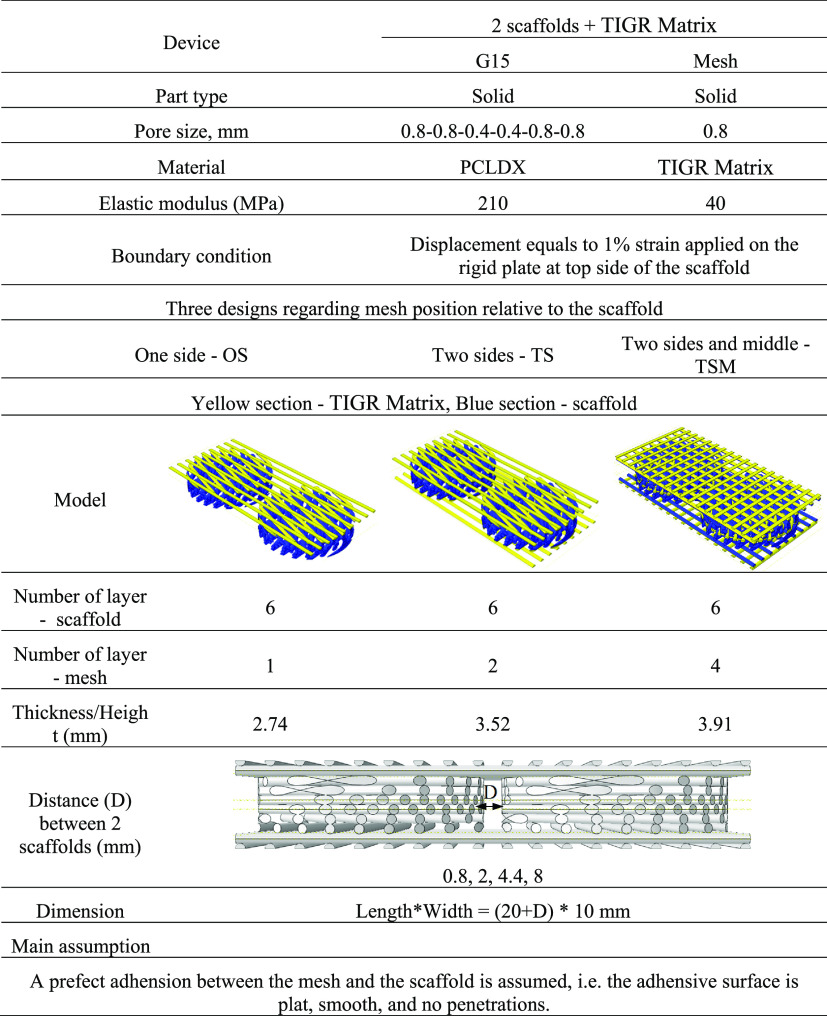
Simulation Case of a Device—Compression

#### 2.10.2 Assessment of Local Stiffness

The spatial distribution of local stiffness represents the stiffness that would be sensed by focal cell attachment points. In total, 65 such attachment points were investigated within a selected area of a single strand. The elastic modulus of PCLDX and different textiles were assigned for the strand. The fabrication method and elastic moduli of different textiles were given in Table S1 in the supporting information. Each individual attachment point covered a segment 1 μm in length, and the stiffness *k*_*L*_ against length change was calculated from the computed reaction force.^38^ It should be noted that the displacements were applied in both normal and shear directions, which represented extreme boundary conditions; hence, cells should be exposed to a stimulus level in the range presented here. To allow the comparison of our results with reported data, the stiffness was then transformed to the Young’s modulus of a 2D plane using the following equation:
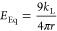
1where *r* is the size of a focal adhesion (1 μm).^39^

### 2.11 Tensile Tests of Textiles

An Instron 5944 (U.S.A.) testing system equipped with a 500 N load cell was used to quantify the tensile properties of the textiles reported in the Supporting Information. The tensile samples were 30 mm long, and depending on the textile, they were 6 mm to 15 mm wide. The samples were elongated at a strain rate of 10% min^–1^ until 50% strain; five replicates of each sample were used, and the average values were reported.

### 2.12 Compression Test of Scaffolds

Scaffold samples (G15, OS, TS, TSM, with TIGR Matrix) 5 mm in diameter were compressed at 37 °C in water using an in-house build testing cell in an Instron 5566 (U.S.A.) testing system equipped with a 500 N load cell. A preforce of 0.1 N was applied, and the scaffolds were compressed by 40% of the original height and a strain rate of 20% min^–1^. The compressive modulus was then calculated from the slope of the stress–strain curve between 0.5% and 1% strain and thus an approximately linear segment of the curve. Five repetitions of the samples were made, and the results are reported as the average ± SD. The quasi-static compressive properties of the G15, OS, TS, and TSM with TIGR Matrix scaffolds were also measured in a climate-controlled room at 50% humidity and 22 °C using 9 mm diameter samples; here three repetitions were made, and the reported values are the average ± SD. Samples of 5 and 9 mm in diameter were equilibrated for 24 h before the aforementioned protocol was applied.

In addition to quasi-static compressive testing, the scaffolds were also studied in 37 °C PBS under cyclic compressive loading. The samples were then compressed 10 times up to 30% strain at a strain rate of 20% min^–1^.

### 2.13 Biaxial Tensile Tests

Planar biaxial testing was used to study the mechanical properties of the G15, TSM, and breast tissue samples using a BioTester (Cellscale, UK) equipped with a 10 N load cell. The samples were merged in a phosphate-buffered saline (PBS) bath at 37 °C to simulate hydration and temperature conditions in vivo. The dimensions of each G15 and TSM sample were 7.5 mm × 7.5 mm, and five repetitions were performed for each subset of samples. Cyclic tests with 10 cycles were performed using a strain rate of 240% min^–1^ until an 18.6% strain was achieved for G15 and 15% for the TSM on both stretch and return. Five samples were tested for the scaffold designs and the average values are shown.

Tissue samples were donated from a healthy patient, who underwent a routine reduction mammoplasty. The samples were transported to the laboratory within 1 h of surgery and placed in PBS at 22 °C. All samples were tested within 8 h of surgery. Fascia within the tissue was isolated, and sections of approximately 10 mm × 10 mm were cut at a thickness of 3 mm. The samples were placed at a distance of 6 mm × 6 mm between rakes. Cyclic tests with 10 repetitions were performed using a strain rate of 10% min^–1^ until 50% strain was achieved on both stretch and return. The data from a representative sample are shown.

For all planar biaxial testing, force/displacement measurements were recorded throughout the test at 10 Hz. Stress–strain curves were generated from these measurements and the original dimensions of the samples. The elastic moduli of adipose tissue samples were calculated as the slope of stress vs strain plots in the first stretch cycle.

### 2.14 Propagation-Based X-ray Phase-Contrast Tomography

The breast tissue sample was imaged using phase-contrast tomography. The imaging relied on propagation-based phase contrast.^40^ The X-ray source was a MetalJet D2 ExAlloy-I1 (Excillum AB, Sweden) operated at 70 kV and 100 W. Images were taken with a sCMOS detector (Photonic Science, UK) with 4096 × 4096 pixels with a pitch of 9 μm, fiber-optically coupled to a 10-μm gadolinium oxysulfide scintillator. The sample was mounted in a plastic tube and imaged with 3001 projections over 360 deg with an exposure time of 3 s per projection. The source-object distance was 230 mm, and the object-detector distance was 390 mm, generating an effective pixel size of 3.34 μm at the object. The images were then postprocessed in software (Exciscope AB, Sweden), which included phase retrieval and tomographic reconstruction using the Feldkamp, Davis, and Kress (FDK) algorithm.^41,42^

### 2.15 Cell Seeding Efficacy

Human dermal primary fibroblast cells (HDFs) (Thermo Fisher Scientific, USA) were cultured in Dulbecco’s modified Eagle’s medium (DMEM) (Thermo Fisher Scientific, USA) supplemented with 10% fetal bovine serum (FBS) and 1% penicillin-streptomycin (Gibco). The cell culture medium was replaced with fresh medium every 3 days until the cells reached 75–80% confluency. The cells were then dissociated using trypsin-EDTA 0.25% (Gibco Thermo Fisher Scientific, U.S.A) and used for experiments (passages 3 an4).

For cell seeding, scaffolds were placed in 48-well tissue culture plates (TCPs), and prior to cell seeding, 0.4 mL of medium was added to the scaffolds, which were centrifuged to remove entrapped air and soaked overnight at 37 °C. The culture medium was then removed from the scaffolds, and a 20 μL cell suspension containing 2.5 × 10^5^ cells was seeded. After 3 h, scaffolds were transferred to another TCP, and 0.5 mL of fresh culture medium was added to the scaffolds and refreshed every 3 days throughout the experiments. Cells not attached to the scaffolds (attached to the bottom of the TCP) were then trypsinized and counted using a hemocytometer, and the seeding efficacy was expressed using the following equation:
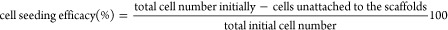
2

Crystal violet (0.1%) (Sigma-Aldrich, USA) dye was used to measure the unattached cells from after cell seeding. Cells were stained by adding the 0.1% dye for 10 min, washed with distilled water to remove excess dye, and visualized using optical microscopy.

### 2.16 Cell Attachment and Proliferation

Cell attachment was assessed at day 1 and proliferation after days 7 and 11 by measuring the DNA content using Picogreen dye (Thermo Fisher Scientific, USA) as described earlier.^43^ Briefly, 0.3 mL of lysate solution was prepared using 0.2 mg mL^–1^ proteinase-k (Sigma-Aldrich, USA) and 0.02% sodium dodecyl sulfate (SDS), added to the scaffolds, and incubated at 37 °C for 12 h. Further, lysate solution was collected, freeze–thawed at −80 °C, and transferred to a 96-well TCP, and an equal amount of Picogreen dye was added to each well and incubated for 5 min. Fluorescence measurements were recorded at 485 excitations and 520 emission wavelengths in a microplate reader. The dsDNA amount was calculated using a standard curve plotted by a serial dilution of a known DNA concentration.

For SEM, cells were fixed on the scaffolds using a 4% formaldehyde solution (VWR, Sweden) for 30 min, and scaffolds were then critically dried using a series of alcohols: 30, 50, 70, 90, and 100%. Confocal microscopy (Zeiss LSM 800) was used to visualize cells in 3D scaffolds. Cells were fixed using 4% formaldehyde, washed with PBS, and stained with fluorescent dyes. Nuclei were stained with SYTOX Green (Thermo Fisher Scientific, USA) in a 1:30000 dilution of stock solution and incubated for 15 min at RT. The cell cytoskeleton was stained using Alexa Fluor 546 dye (6.6 μM; 30 min; Thermo Fisher Scientific, USA).

## 3 Results and Discussion

### 3.1 Mechanical Properties of Breast Tissue

**Figure 2:**
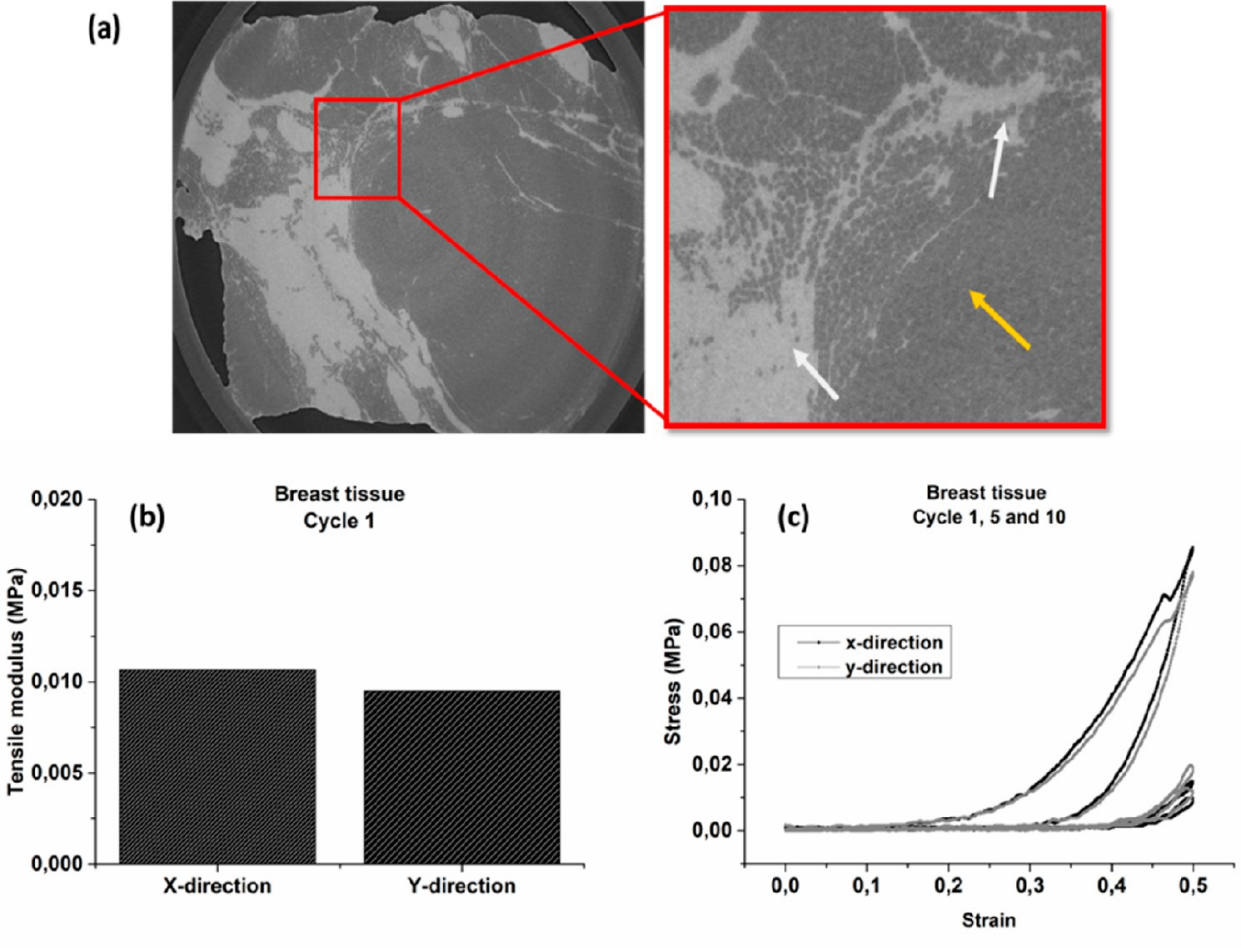
(a) μCT images of the heterogeneous adipose tissue with the inset and white arrows showing the fibrous fascia while the yellow arrow shows the adipocytes; (b) tensile modulus of a sample of heterogeneous adipose tissue in the first stretch cycle; (c) Cauchy stress variation in the *x* and *y* directions during cycles 1, 5, and 10.

Native breast tissue is a highly heterogeneous structure composed of a high degree of adipose tissue, as well as glandular tissue and the superficial fibrous fascia.^28,29^ This fibrous structure, which forms a 3D network in the breast often denoted as suspensory ligaments or Cooper’s ligaments, develops both as thicker bundles to suspend the breast and as fine, hair-like fibers that infiltrate through the adipose tissue region. This fibrous part of the tissue provides structural support to the soft adipose tissue. Figure 2 shows μCT images of the fibrous fascia and data from planar biaxial testing. The heterogeneous distribution of the fibrous tissue was observed (white arrows in Figure 2a) together with mature lipid-filled adipocytes (yellow arrows in Figure 2a).

The breast tissue sample was biaxially deformed in a cyclic manner. The tensile moduli of the sample at 20% strain and the variation in stress during test cycles 1, 5, and 10 are shown in Figure 2 panels b and c. Both the stiffness and stress of the breast tissue sample tested showed isotropic properties with respect to the imposed strain in the *x*–*y* direction. This behavior is similar to what has been seen for pure adipose tissue when it has been modeled as an isotropic closed cell foam.^44^ In addition, the elastic modulus found in tension and compression has been seen to be comparable. The tensile modulus of the breast tissue sample herein has a value of 0.01 MPa (Figure 2b), closely approximating values of fibrous breast tissue measured by others; however, the exact value was highly dependent on the precompression strain.^45^ The measured tensile modulus of the breast tissue was higher than previous published data of the elastic modulus of pure adipose tissue, indicating the presence of fibrous tissue in the sample.^31,45^ The presence of fibrous tissue can be seen in the μCT tomographs indicated by the white arrows in Figure 2a. The effect of the cyclic load shows that the fatigue behavior results in a reduction of the maximum stress to a level of approximately 0.01 MPa within a few load cycles, as shown in Figure 2c. Anisotropic behavior, depending on the directionality and composition of collagen fibers, has been observed by other researchers in *ex vivo* biaxial mechanical testing of adipose tissue without any fixation, such as formalin.^33,46^ It should be noted that the absolute values depend on the dissected section of the adipose tissue. Thus, the content of the fascia in the sample depends on the location in the breast tissue, patient, and the test condition. The intention with this mechanical testing of the tissue was not to give an absolute value but to understand the response of the tissue to mechanical deformation and hence to design a scaffold that would aid the regeneration of the missing tissue.

### 3.2 Mechanical Properties and Simulations of the Modular Scaffold

**Figure 3:**
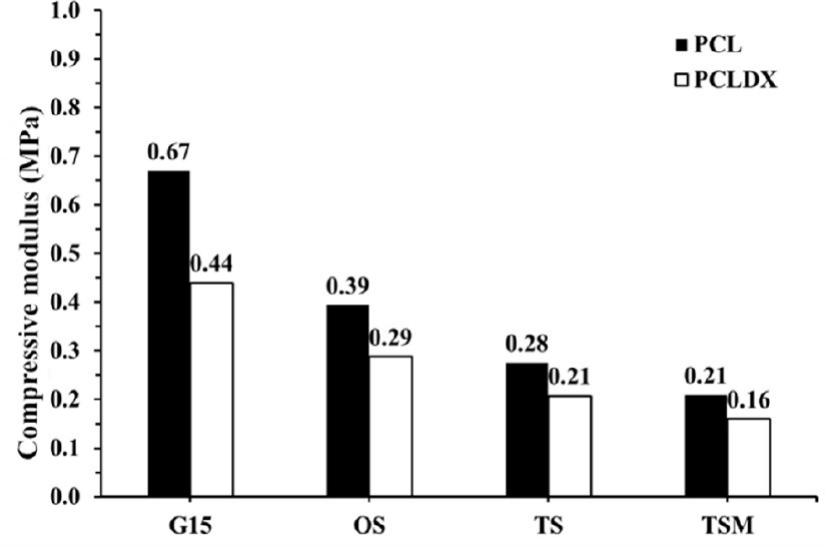
Simulation of the compressive modulus of the modular scaffold designs G15, OS, TS, and TSM as well as influence of the polymer of the printed scaffold PCL vs PCLDX.

As previously mentioned, PCL is becoming one of the standard materials used in tissue engineering.^12,14,47^ It has been of interest also for breast reconstruction, but has an unsuitably long degradation time for this use. It was therefore used as a control in the simulation of G15, OS, TS, and TSM to see how PCLDX would influence the mechanical properties of the modular combined design to compare the results more easily to the literature, Figure 3.

**Figure 4:**
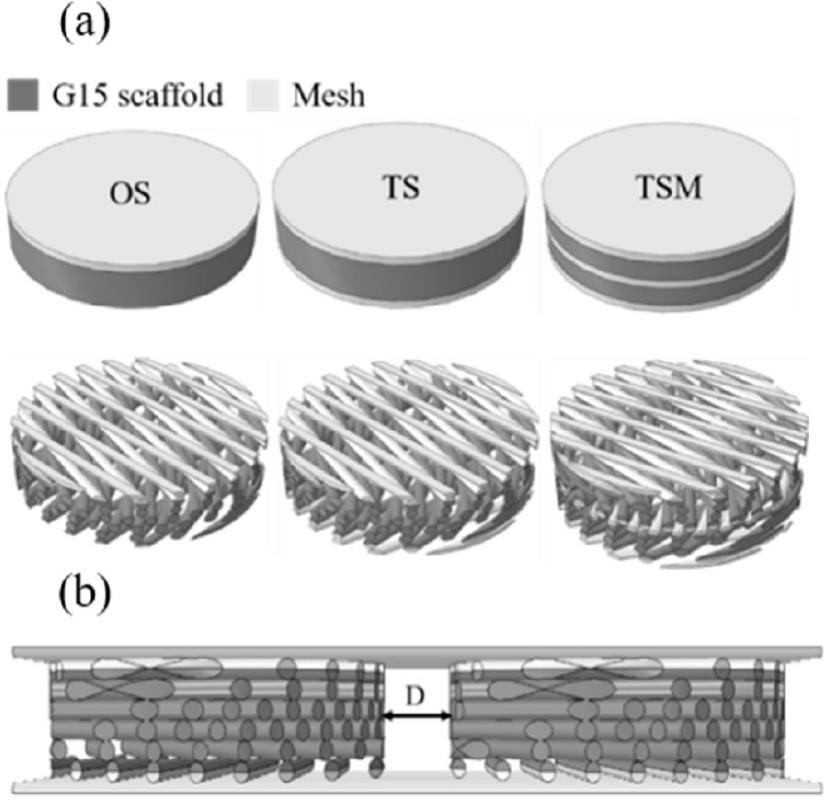
Computer aided design files (a) and the prototype of two scaffolds connected by two TIGR Matrix strips (b). D denotes the distance between the two units.

As shown in Figure 3, the addition of the knitted mesh with the printed structures would not only produce a thicker scaffold that could potentially increase the amount of tissue compared to a thin mesh, but also represent a possible way to adjust the mechanical properties by reducing the compressive modulus. The more mesh that is added to the design, the more the damping ability increases, and the more energy is absorbed by the scaffold unit. The use of PCLDX in the G15 design and the TSM design results in a reduced compressive modulus as low 0.16 MPa, with these values being comparable to experimental values of breast tissues and in line with what others have stated are in the needed range (fibrous and normal glandular tissues).^14,31^ Additionally, the compression simulation results revealed that the computed maximum von Mises stress within the modular scaffolds significantly decreased from 40.4 (G15) to 18.2 (OS), 16.3 (TS), and 11.6 MPa (TSM), such that the more layers of mesh are added, the less energy due to compression is absorbed by the scaffold unit. The influence of the mechanical properties of the mesh layer have been further explored using knitted polylactide textiles (Figure S1 and Table S1 in the Supporting Information). Simulation results in compression and tension can be viewed in Figure S3 and Figure S4, as well as the influence of pore size in the G15 design in Figure S5. The benefit of using a modular design approach with textiles is that one scaffold unit can be joined together using the mesh to form larger devices of low stiffness, Figure 1. Depending on the distance (*D*) separating two scaffolds, the compressional modulus for a larger device can be controlled, Figure 4.

If *D* between two scaffolds is increased from 0.8 to 8 mm a decreased compressive modulus for devices was seen for OS (from 0.22 to 0.16 MPa), TS (from 0.18 to 0.13 MPa), and TSM (from 0.17 to 0.12 MPa), Figure 4 and S6 in the Supporting Information. The simulation of the difference in compression reveals that a lower stiffness can be obtained for devices with a larger size by controlling the layout of the modular scaffold units.

### 3.3 Local Stiffness and Simulation

**Figure 5:**
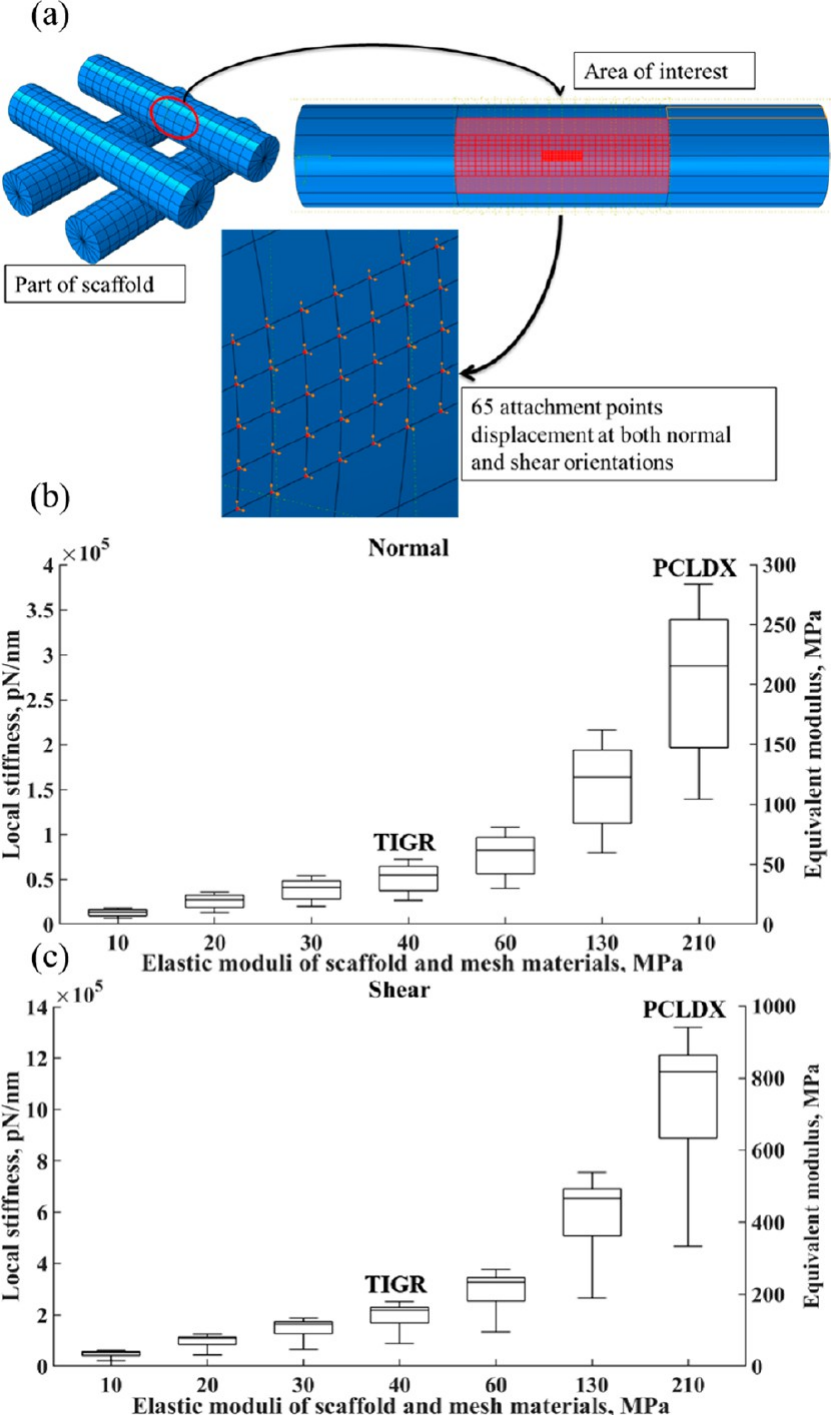
Simulation results of local stiffness (a) show a visual overview of how the simulation of local stiffness was carried out and two box plots reporting both the local stiffness and the equivalent modulus for a selected strand area shown in panel a within the scaffold depending on the elastic modulus of the material used (b) calculated in the normal direction and (c) the shear direction to the strand.

The compression simulation results provide knowledge of how to design a scaffold with mechanical properties suitable for adipose tissue regeneration. Including our previous results, which showed that the G15 design has a homogeneous distribution of stress and few stress bridges,^11^ we believe that the design is also able to shield the cells in the interior against external deformation. However, describing the mechanical environment that cells experience in a 3D porous scaffold is still a challenging task. The mechanical stimulus that cells receive from a 2D substrate is the stiffness, which depends on the material properties. However, within a porous surface in a 3D structure, it is the local stiffness that defines the mechanical properties that cells experience, which depends on both the material properties and the pore topography.^38^ Experimental measurements of the local stiffness within a porous substrate, curved surface, by using atomic force microscopy are difficult to conduct.^48^ We therefore carried out a simulation study to predict the local stiffness in a selected strand surface area within the scaffold unit both perpendicularly to the strand, denoted as normal, and adjacent to the strand, denoted as shear, Figure 5. Here, the mechanical stimulus due to cell traction on the surface was predicted by simulating the deformation generated from a boundary condition of a unit micrometer displacement.

The simulation results demonstrate a nonuniform distribution of local stiffness within the selected surface, Figure 5b,c. The median local stiffness ranged from 1.7 × 10^4^ to 2.8 × 10^5^ pN/nm in the normal orientation and 4.1 × 10^4^ to 11.2 × 10^5^ pN/nm in the shear orientation when the strand materials increased from 10 to 210 MPa. A clear difference in the local stiffness distribution was observed between the shear and normal orientations to the strand surface. These observations indicate that the local stiffness depends not only on the material properties but also on the cellular orientations. In addition, the equivalent modulus calculated from the computed local stiffness is summarized in Figure 5b,c. The median equivalent modulus ranged from 23.4 to 818.8 MPa in shear and from 11.1 to 215.2 MPa in the normal orientation when the substrate materials increased from 10 to 210 MPa. The results suggest that the local mechanical stimulus generated by the strand surface is controlled by the elastic modulus of the polymer rather than the scaffold architecture. This finding, in turn, shows that simply reproducing the bulk mechanical properties of replaced tissues may not be enough for a scaffold. The pore structure and the polymer used to print the scaffold play a key role in generating the local mechanical stimuli to which cells are sensitive. Given our simulation showing the influence of the elastic modulus on the equivalent modulus and local stiffness, Figure 5, our design shows great potential to cover a wide range of compression and tensile moduli from hundreds of kPa to tens of kPa, which matches the mechanical properties of breast tissue.^31^ By using a modular scaffold design with different hierarchical components there are a wide range of possibilities available for adjusting the cell-material interaction in comparison to a 3D–printed scaffold alone.

### 3.4 Modular Scaffold Fabrication

The G15 scaffold was printed using FFF, and the parameter settings used during filament spinning and printing as well as the characterization results of the thermal properties of the polymer before and after each manufacturing step were determined through DSC and SEC and are shown in Table 4.

**Table 4:** Characterization of the PCLDX Granules, Filament, and Scaffold through DSC and SEC after Each Production Step. The Parameters Used at Each Production Step Are Also Listed

PCLDX	*T*_m_ (°C)^a^	*X*_c_ (%)^a^	*M*_n_ (kg mol^–1^)^b^	Đ^b^	Extr. temp(°C)^c^	Extr. speed (rpm)^c^	Print temp (°C)^d^	Print speed (mm/s)^d^	Fan speed (%)^d^
Granules	44	34	102	2	145	9			
Filament	51	35	110	2.15			170	5	100
Scaffold	46	32	116	2.1					

a Reported from the first heating run of the DSC cycle.

b Characterization by SEC in CHCl_3_. Calibration was performed using polystyrene standards with narrow dispersity. The average values of three samples are reported

c The parameters used in the Felfil evo filament maker.

d The parameters used in the Prusa i3MKS.

**Figure 6:**
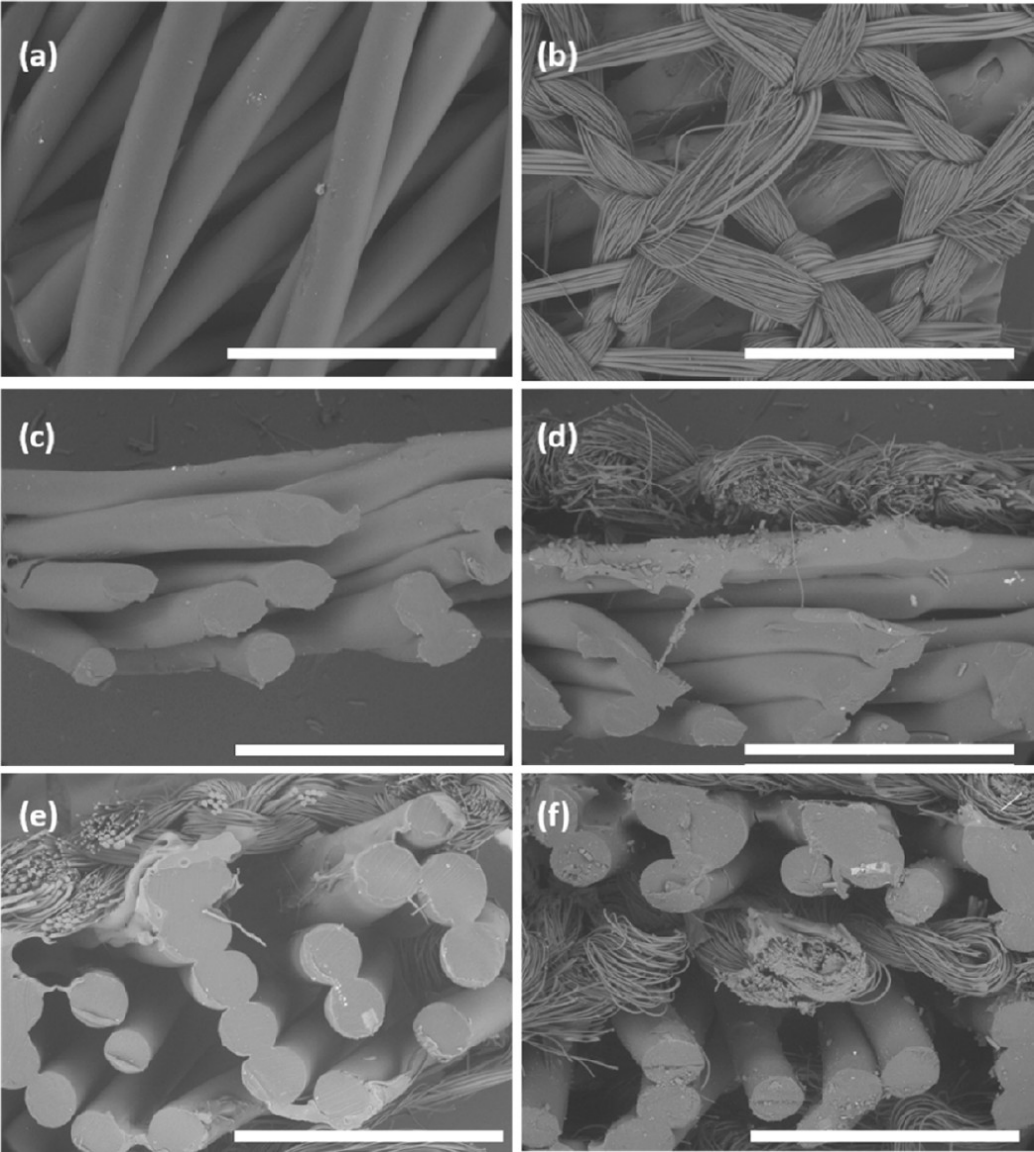
Micrographs show modular scaffold units as seen from above (a), the TIGR Matrix attached using the degradable adhesive on a scaffold from above (b), the G15 design (c), the OS design (d), the TS design (e), and TSM from a cross-sectional view (f). All micrographs were taken at 50× magnification, and the scale bars correspond to 2 mm.

The characterization of the polymer before and after each manufacturing step in Table 4 shows that the thermal properties and molar mass of the scaffolds are very similar to those of the granules. The printing temperature is higher than the extrusion temperature due to the smaller nozzle size of the printer but is reduced in comparison to what has previously been used for this polymer due to the combination of a lower printing speed, a section of a printer with a shorter distance between the feeding gear and nozzle as well as increased cooling at the printing head.^9^ After assembly of the modular scaffolds of the 3D-printed G15 design with the design’s tabletop, SEM images were taken from above and cross-section, Figure 6.

**Figure 7:**
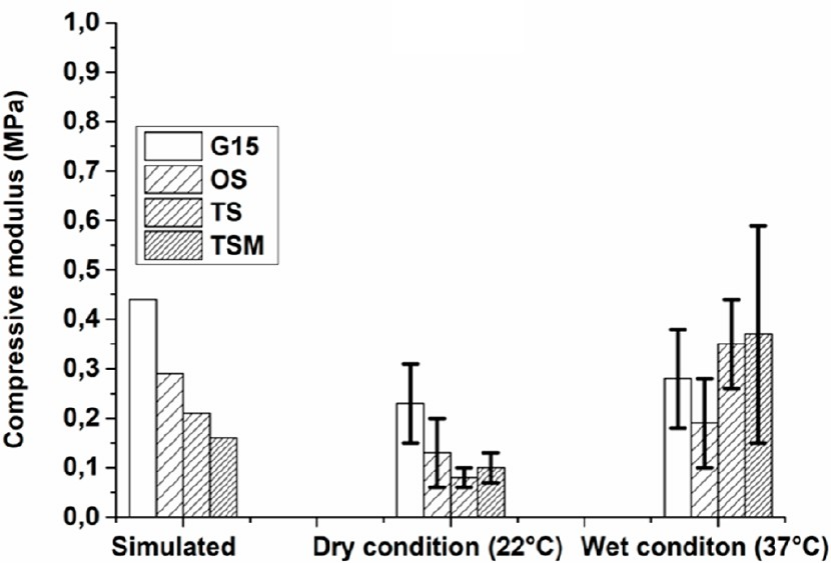
Simulated compressive modulus of G15 and OS, TS, and TSM designs are shown together with experimental data from dry and wet conditions. The data of the dry conditions tests are reported as the average of three repeats ± SD. The data of the wet conditions tests are reported as the average of 5 repeats ± SD.

The micrograph of the modular scaffold design in Figure 6 shows the similarity to the CAD models in Figure 4. The average strand thickness of 0.4 mm correlates well to the nozzle size of 0.4 mm; however, the distance between two strands is wider, up to 1 mm, in comparison to the intended design of 0.8 mm. The SEM images of the top and cross-sectional view of the scaffold combination in Figure 6 shows that the TIGR Matrix is placed directly on the scaffold and that the adhesive has not penetrated the structure and is not blocking the pores either from the top sectional view or from the cross-sectional direction. Additionally, the strands are evenly sized, and the geometry conforms to the intended design. To validate the simulation of how the assembled modular design and the introduced meshes affected mechanical properties, experimental tests were conducted in wet and dry conditions, Figure 7.

**Figure 8:**
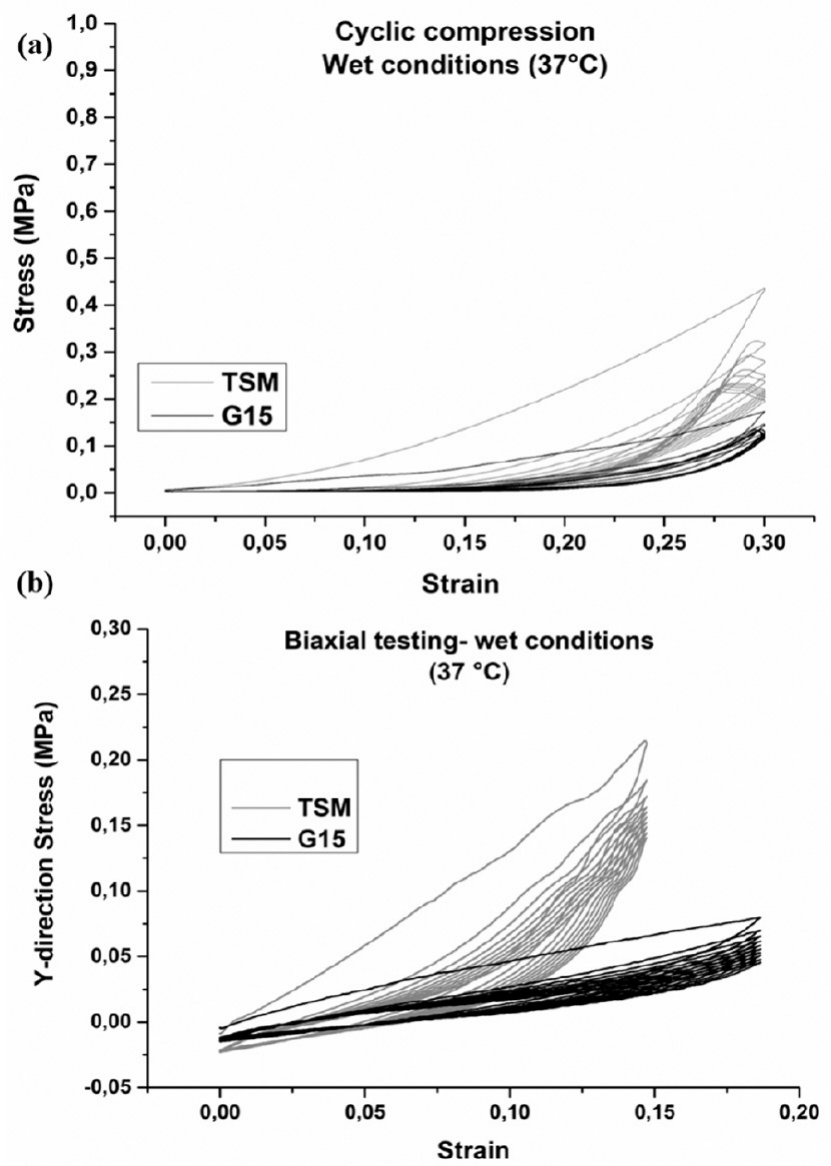
G15 and TSM designs are shown from (a) cyclic compression testing in PBS at 37 °C (b) and biaxial tensile testing in the *y*-direction over 10 cycles. The average values of five samples are shown.

The simulation results showed that the introduction of more layers of mesh in the modular design resulted in a lower elastic modulus (G15 > OS > TS > TSM), as shown in Figure 7. The same tendency can be observed in the experimental results in the dry state 7. The adhesion section between the scaffold and the mesh was not considered in the FEA simulations. However, this adhesive layer may play a role in the mechanical response of the modular scaffold unit under compression testing, which could be the reason for the discrepancy between the simulated results and the measured data with the high error bars. While testing in 37 °C in PBS resulted in a large variation in the elastic modulus, the compressive stiffness was still in the range of the FEM-based results. Theoretically, the wet state at body temperature would reduce the scaffold elastic modulus compared to the dry state at room temperature.^49^ However, this trend is not seen in Figure 7. This difference can be attributed to the addition of an adhesive layer that may swell in the wet state resulting in increased stiffness.^36^ To understand how fatigue behavior is influenced by scaffold design, the TSM and G15 designs were tested in cyclic compression and biaxial tension, Figure 8.

Figure 8a and b illustrate a significant hysteresis at cycle testing (both compression and biaxial tensile testing), and the stress response decreased after the first load cycle. In subsequent cycles, the stress–strain curves show stiffening with increasing strain behavior, and the loading and unloading paths become similar. This observation in the stress–strain curves is in accordance with previous research.^50^ The ability to recover after deformation plays a crucial role when using polymers in soft tissue engineering scaffolds and depends on the deformation behavior of the polymer as well as the 3D scaffold structure. The 3D architecture of scaffolds may experience bending after initial loading, which in turn prevents or facilitates the strands to move back to their original positions. As seen from the compression tests in Figure 7, the TSM design had a large SD, thus causing the 10th cycle to be not significantly different from the G15. Both scaffold designs were found to be isotropic in the biaxial tests; the *x*-direction values can be viewed in Figure S7. The absolute values of the biaxial tests in Figure 8b cannot be directly compared to the breast sample data in Figure 2c due to difference in strain rate, as well as variations of the superficial facia and the interference from the adhesive, it is however observed that they are in the same range.

### 3.5 Cell Material Interactions

**Figure 9:**
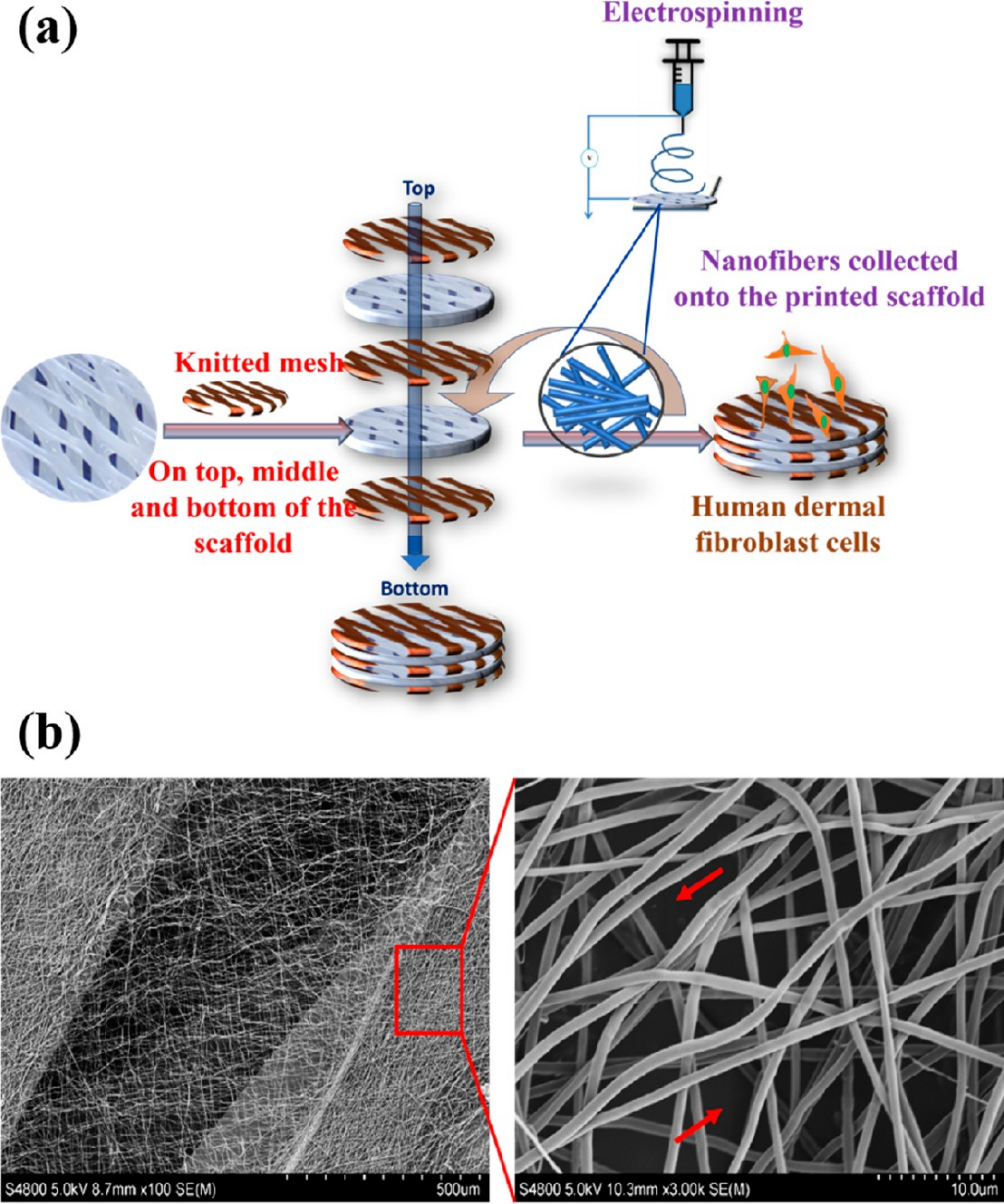
(a) Assembly process of the TSM_NF design is shown in; (b) SEM micrograph of electrospun nanofibers collected on the G15 scaffolds (red arrows): (left) scale bar = 500 μm; (right) scale bar = 100 μm.

*In vitro* cell material interactions were assessed to understand whether cells adhere and proliferate on and inside the scaffold unit. On the basis of the FEA results and experimental evaluation of the mechanical properties in the TSM design, the addition of knitted mesh lowered the modulus. Therefore, TSM design was considered further for the *in vitro* assessment. The modular architecture in TSM provides a larger surface-to-cell attachment compared to the G15 design alone, but the pores presented in TSM are too large to easily facilitate cell adhesion, Figure 6. If the pore size is larger than the cells, they will pass through the direct pores and thereby cause a low cell seeding efficacy. However, a large pore size is needed to aid the access of nutrients within large devices, and if the pore size is less than 400 μm, it has been shown to hinder angiogenesis, limiting adipogenesis.^51^ To overcome this issue while maintaining porosity, the TSM design was further modified by including an electrospun nanofiber layer. Of interest, though not evaluated here, is that we saw from FEA simulations in Figure 5b,c that the mechanical properties of the intermediate layers would influence the local stiffness distribution and magnitude. Electrospun layers in which the fibers are in the size range of the ECM environment might therefore also contribute to an internally favored microenvironment for 3D printed structures by lowering the local stiffness.^19,52^ The nanofibers were collected directly onto the 3-layer G15 scaffold replacing the middle TIGR Matrix layer in the TSM design, named TSM_NF. Figure 9a shows the assembly of the TSM_NF scaffold and Figure 9b shows SEM micrographs of nanofibers on the G15 scaffolds.

**Figure 10:**
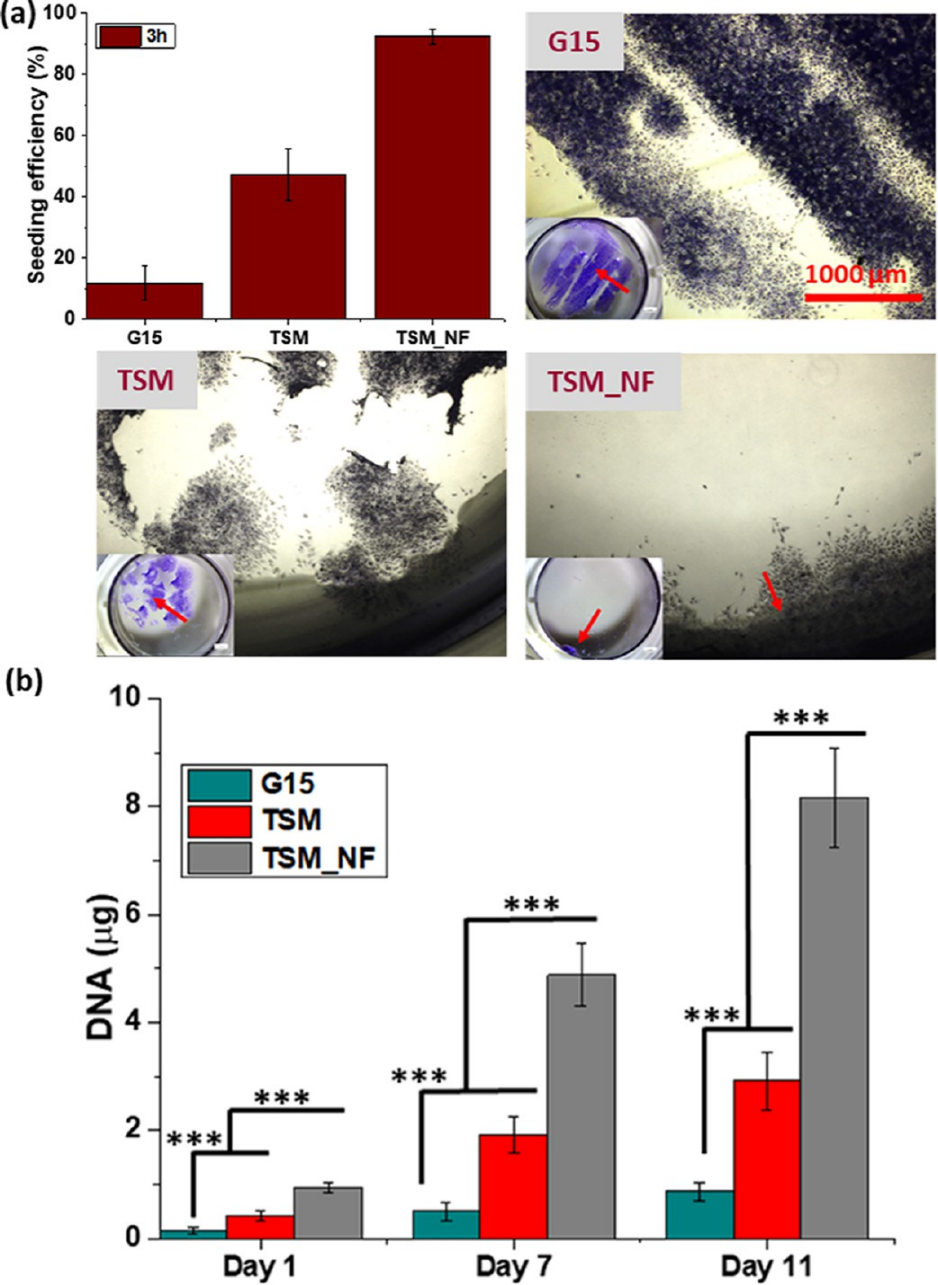
Influence of the scaffold design on the seeding efficacy. (a) Seeding efficiency was determined after 3 h, and optical images (4×; scale bar = 1000 μm) of unattached cells stained with crystal violet on G15, TSM, and TSM_NF scaffolds were obtained. The effect of pore geometry can be seen in the optical images of the TCP plates, where the number of cells attached, and the features of the pores can be seen. The inset shows (scale bar = 1 mm) an image of the complete TCP well after cell seeding. (b) DNA quantification of the cells cultured on G15, TSM, and TSM_NF on days 1, 7, and 11. Significant differences are shown by the symbol *** *P* < 0.001 and presented as ± SD.

The SEM micrographs in Figure 9 show that the nanofiber morphology was homogeneous without any beads, and their diameter was in the 700–800 nm range. We further evaluated the cell-material interaction of the TSM, TSM_NF, and G15 which was used as a reference. HDFs were used, and cell seeding, attachment, and proliferation were evaluated. Cell seeding efficiency was determined after 3 h, Figure 10a. Unattached cells on TCP were stained with crystal violet and visualized utilizing optical microscope, Figure 10a. The DNA content of the cells cultured on G15, TSM, and TSM_NF on days 1, 7, and 11 was also quantified to assess attachment and proliferation, Figure 10b.

The results in Figure 10a show that the G15 alone had a significantly lower seeding density (≈12%) than TSM (≈47%), and the highest seeding density was seen in TSM_NF (≈92%). The G15 had larger pores, resulting in fewer attachment points for cells, whereas TSM provided an extra surface with multiple layers that significantly increased cell adhesion. Despite the multilayer architecture of the TSM, almost half of the cells passed through the pores, attributable to their larger pore size needed to achieve vascularization in a larger device and gaining the bulk mechanical properties intended. However, in TSM_NF, cells passing through the pores of the mesh and 3D-printed G15 design adhered to the nanofibers. The nanofiber network increased cell seeding efficacy, thereby increasing cell adhesion and proliferation in the scaffold.^53^ For a larger device implanted *in vivo* the nanofibers would contribute to the increased surface area of the device due to their high surface-to-volume ratio and would allow nutrient exchange mimicking extracellular matrix fibers. In addition, nanofibers can be functionalized with the addition of peptides to promote vascularization or reduce inflammatory response after implantation.^22^ To further support the results, optical photographs were taken of the TCP, showing the effect of the scaffold design Figure 10a. It was seen that cells passed through the pores of the G15 and TSM design, while fewer cells were seen doing so with the TCP with the added nanofibers, and TSM_NF scaffolds.

**Figure 11:**
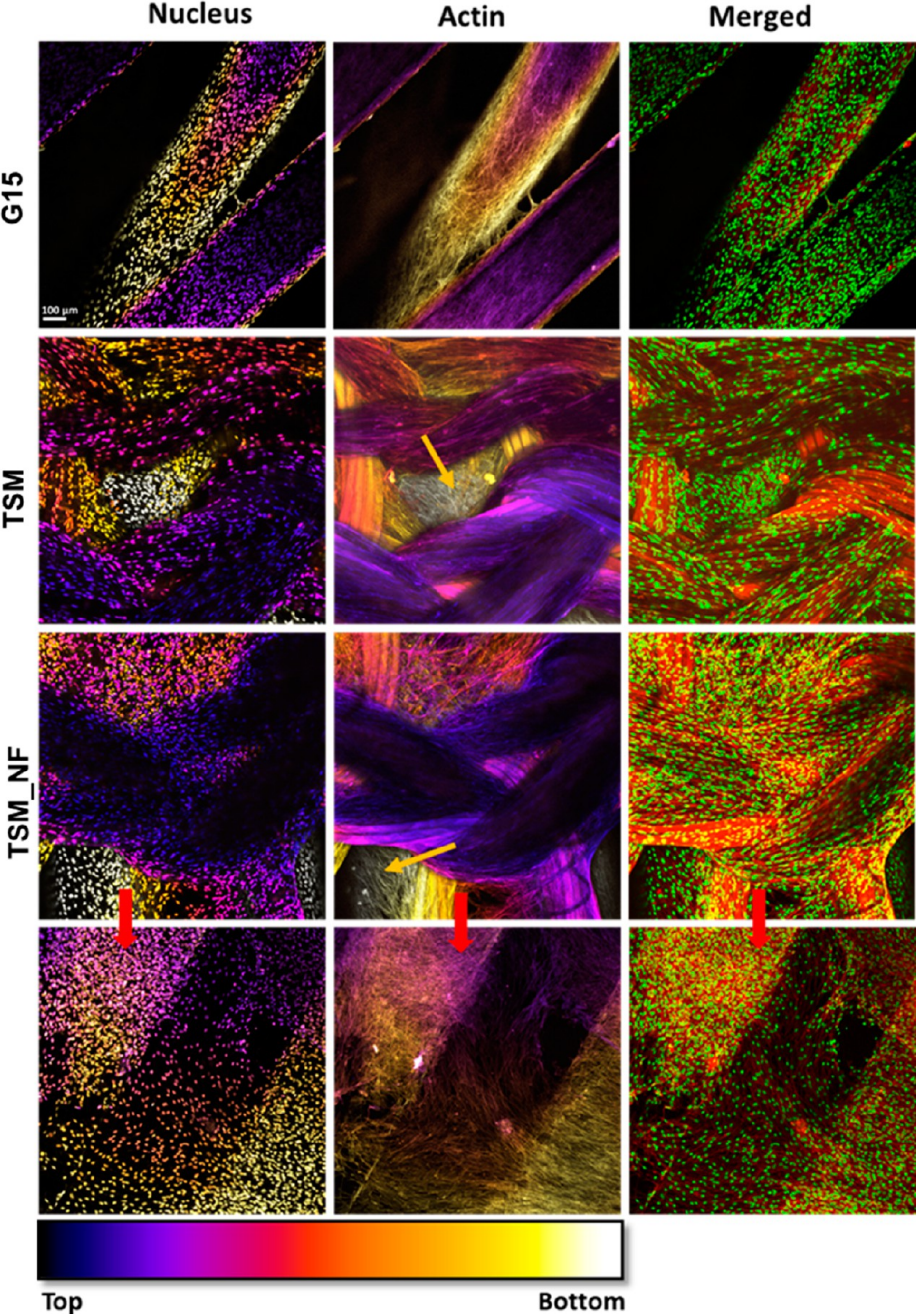
Confocal micrograph at day 7 showing cell distribution on and inside the G15, TSM, and TSM_NF scaffolds. The yellow arrow shows the cells on the 3D-printed scaffold in the modular designs. One mesh layer and a three-layered 3D-printed structure were gently removed from the TSM_NF design to see the nanofiber network. The red arrows show the cells attached to the nanofibers in the TSM_NF scaffolds. Color code representation was assigned using ImageJ. (Scale bar = 100 μm).

On day 1, cell attachment was measured by quantifying DNA content that can be directly correlated with cell numbers Figure 10b. The results showed that cells were attached and spread on all scaffolds, and G15 had significantly lower DNA than TSM. Similarly, TSM_NF had the highest measured DNA content Figure 10b. This can be attributed to the initial cell-seeding efficiency, which was higher in TSM_NF than in the G15 and TSM scaffolds. We further assessed cell proliferation on days 7 and 11. The results showed that in Figure 10b, all scaffolds supported cell proliferation, which significantly increased from day 7 to 11. A similar trend was observed on days 7 and 11 in cell proliferation. The DNA content was higher in TSM_NF than in TSM, and the G15 had the lowest DNA content, which was expected given their differences in initial cell adhesion due to scaffold design and insertion of nanofibers in the TSM_NF design. Nanofibers have a greater surface area that aids in cell adhesion and faster proliferation. The cell proliferation was further visualized using confocal microscopy at 7 days, Figure 11.

On day 7, cells were observed to cover almost the nanofiber layer, suggesting higher cell proliferation, Figure 11, which increased further on day 11 and when the scaffold was fully covered by cells. These initial cell study results suggest that the combination of 3D-printed scaffolds, knitted mesh and electrospun nanofibers significantly improves cell material interaction performance in terms of both initial cell adhesion and proliferation compared to the G15 design. This is caused by the introduced layers of knitted mesh and electrospun nanofibers with reduced pore size that provide extra surface area and more attachment points for cells. Further SEM analysis of cell proliferation onto the scaffold designs are shown for day 7 and day 11 in Figure S8. From the FEA simulation, we observed that these intermediate layers would influence the local stiffness distribution and magnitude, which in turn generate local mechanical stimulus at different levels that cells may experience. However, further study is needed to evaluate how the different local stiffnesses of each construct influence the cell in detail.

## 4 Conclusion

Designing a modular scaffold and tuning the mechanical properties at both the macroscale and microscale is crucial for providing sufficient external mechanical support and creating an internal cell-like environment to support breast tissue regeneration.

We propose a modular scaffold design TSM_NF combining the 3D-printed G15 design which provides volume and compressional strength, with knitted mesh and electrospun nanofibers. The deformation response of the modular design scaffolds was assessed by experiments and FEA simulations. The FEA simulation showed a reduction in compressional stiffness with the introduction of mesh layers through the TSM design, seen in the experimental tests under dry conditions but not identified under wet conditions at 37 °C due to the influence of the applied adhesive. It does, however, improve the cell seeding efficacy and opens the possibility of creating a larger device such as an internal bra without increasing the stiffness of the construct or reducing the pore size of the 3D printed structure preventing the ingrowth of new tissue. In addition, the predicted spatial stiffness distribution indicated that cells are exposed to a heterogeneous local mechanical stimulus, and the magnitude of such mechanical stimuli was found to be determined by the scaffold material rather than the scaffold structure. Furthermore, initial cell studies showed that modular scaffolds, including knitted mesh and electrospun nanofibers, possess better performance in terms of cell seeding, cell attachment, and proliferation than 3D-printed G15 scaffolds alone. Thus, it can be concluded that the design concept presented here provides a promising method for soft tissue engineering scaffolds to assist in breast reconstruction.

## Supporting Information Available

Micrographs of the additional textiles used for simulation; description and tensile modulus of the textiles used for FEA simulation; description of the FEA simulation cases in compression; FEA simulation of compressional modulus with varying elastic moduli of mesh; description of the FEA simulation cases in tension; FEA simulation of designs in compression; FEA simulation of designs in tension; FEA simulation of G15 in tension with varying pore size; FEA simulation of a device using the designs in compression with varying distances; biaxial testing of TSM and G15 in *x*-direction; SEM micrographs of proliferation of cells onto the designs, yellow arrows showing attached cells (PDF)

## References

[ref1] Sung H.; Ferlay J.; Siegel R. L.; Laversanne M.; Soerjomataram I.; Jemal A.; Bray F. Global Cancer Statistics 2020: GLOBOCAN Estimates of Incidence and Mortality Worldwide for 36 Cancers in 185 Countries. Ca-Cancer J. Clin. 2021, 71 (3), 209–249. 10.3322/caac.21660. 33538338

[ref2] Combellack E. J.; Jessop Z. M.; Naderi N.; Griffin M.; Dobbs T.; Ibrahim A.; Evans S.; Burnell S.; Doak S. H.; Whitaker I. S. Adipose regeneration and implications for breast reconstruction: update and the future. Gland Surg. 2016, 5 (2), 227–241. 27047789 10.3978/j.issn.2227-684X.2016.01.01 PMC4791352

[ref3] Potter S.; Conroy E. J.; Cutress R. I.; Williamson P. R.; Whisker L.; Thrush S.; Skillman J.; Barnes N. L. P.; Mylvaganam S.; Teasdale E.; Jain A.; Gardiner M. D.; Blazeby J. M.; Holcombe C.; Achuthan R.; Adwan I.; Aggarwal S.; Ahmed M.; Akelund M.; Akolekar D.; Al-Jibury O.; Amanita M.; Appleton D.; Archampong D.; Asgiersson K.; Athwal R.; Augusti A.; Ayaani S.; Bains S.; Baker B.; Baker E.; Baldota S.; Banerjee D.; Barker S.; Barr L.; Barry P.; Basu N.; Bathla S.; Bishop N.; Boland G.; Branford O. A.; Bright-Thomas R.; Brindle R.; Brock L.; Brown V.; Bux F.; Byrne G.; Cain H.; Caldon L.; Callaghan M.; Carbone A.; Carpenter R.; Cawthorn S.; Chagla L.; Challoner T.; Chalmers C.; Chalmers R.; Chambers S.; Chana M.; Chand N.; Chandran V.; Chandrashekar M.; Charfare H.; Chatterjee J.; Chatterjee S.; Chattopadhyay R.; Chaudry A.; Chin K.; Chong K.; Chouhan A.; Choy C.; Christopoulos P.; Clarke D.; Clarke S.; Clayton E.; Clifford R.; Cocker D.; Collin T.; Collis N.; Conroy F.; Constantinou C.; Conway A.; Cook J.; Coombs N.; Cox K.; Critchley A.; Dakka M.; Dani M.; Daoud R.; Darragh L.; Darvesh S.; Dash I.; Datta S.; Davies E.; Dawson S.; De Sousa E.; Debnath D.; Deol H.; Devalia H.; Di Micco R.; Dicks J. R.; Dickson J.; Dobner N.; Dobson G.; Dunne N.; Egbeare D.; El Sharief D.; Elfadl D.; Eltigani E.; Enver D.; Erel E.; Evans A.; Exarchos G.; Fage E.; Fatayer H.; Fenn C.; Ferguson D.; Foulkes R.; Franks J.; Fung V.; Galea M.; Gandamihardja T.; Gandhi A.; Garnsey C.; Gateley C.; Gattuso J.; Gawne S.; Geerthan N.; Ghattura A.; Giaramadze A.; Gill J.; Godden A. R.; Goh S.; Govindarajulu S.; Goyal S.; Graja T.; Granger S.; Green M.; Grover K.; Gui G.; Gurung R.; Gutteridge E.; Hakim A.; Halka A.; Hamilton-Burke W.; Hamo I.; Harding-Mackean C.; Hargreaves A.; Harries S.; Harris K.; Harris P.; Harrison S.; Harvey J.; Hashem M.; Hassan U.; Henderson J.; Henton J.; Hignett S.; Hodgkins K.; Horgan K.; Horn S.; Hu J.; Hussain A.; Iddon J.; Iqbal A.; Irri R.; Irvine T.; Irwin G.; Iskender A.; Ismail A.; Ives C.; James K.; James R.; Jiwa N.; Jobson M.; Joglekar S.; Johnson L.; Johnson R.; Johnson R.; Jones L.; Ju Hwang M.; Kalles V.; Kanesalingam K.; Karat I.; Kaushik M.; Kennedy K.; Khalifa E.; Khan H.; Khanbhai M.; Khawaja S.; Khout H.; Kiernan T.; Kim B.; Kirkpatrick K.; Kiruparan P.; Kirwan C.; Kishore M.; Kneeshaw P.; Knight A.; Kohlhardt S.; Krupa J.; Krupa K.; Kuruvilla R.; Laban C.; Lai L. M.; Laidlaw I.; Lambert K.; Langlands F.; Lansdown M.; Laurence N.; Laws S.; Ledwidge S.; Lefemine V.; Lennon H.; Linforth R.; Little K.; Luangsomboon A.; Lund J.; Maalo J.; MacLennan L.; Macmillan R. D.; MacNeil F.; Mahapatra T. K.; Mallidis E.; Mallon P.; Manoloudakis N.; Maraqa L.; Marla S.; Masood S.; Massey J.; Masudi T.; Matey P.; Mazari F.; McCulley S.; McEvoy K.; McIntosh J.; McIntosh S.; McKenzie S.; McManus P.; McNicholas J.; Michalakis I.; Mills N.; Mitchell G.; Monib S.; Mullan M.; Murphy C.; Murphy G.; Murphy J.; Murthy B.; Musa S.; Nagra G.; Nangalia R.; Narayanan S.; Nasr R.; Navin C.; Newton R.; Nicholson S.; Nuru N.; O’Connell R.; O’Donoghue J.; Ogedegbe A.; Olayinka O. S.; Olsen S.; Osborn G.; Osborne C.; Osman H.; Otieno C.; Pakzad F.; Park A.; Parker S.; Partlett P.; Parvaiz A.; Parvanta L.; Patel G.; Peel A.; Peiris L.; Pennick M.; Peppe A.; Perry D.; Pilgrim S.; Piper J.; Poonawalla S.; Popa E.; Pope V.; Pugh P.; Rainsbury D.; Ramsey K.; Rasheed T.; Rathinaezhil R.; Rattay T.; Ravichandran D.; Reed M.; Refsum S.; Remoundos D.; Rigby K.; Robertson S.; Robinson A.; Robinson J.; Roche N.; Roy P. J.; Runkel M.; Rusby J.; Saha S.; Saidan Z.; Salab M.; Saleh M.; Salem F.; Sami A.; Samlalsingh S.; Sarfraz N.; Shah R.; Shaheed S.; Sharaiha Y.; Shetty G.; Shotton R.; Sircar T.; Skene E.; Sloan S.; Smith B.; Smith J.; Soldanova L.; Soliman F.; Soumian S.; Stevens J.; Steventon C.; Stewart-Parker E.; Stringfellow T.; Sutaria R.; Sutton R.; Sweetland H.; Swiech B.; Tadiparthi S.; Tafazal H.; Taheri N.; Tait C.; Tan M.; Tang S.; Tansley A.; Tate S.; Tayeh S.; Taylor A.; Taylor J.; Thawdar P.; Thomas C.; Thomas S.; Thomson S.; Thorne A.; Tillett R.; Tolkien Z.; Tomlins A.; Topps A.; Tsang F.; Turner E. J.; Turton P.; Udayasankar S.; Ugolini F.; Vaughan Williams E.; Vidya R.; Vijaynagar B.; Vinayagam R.; Volleamere A.; Voynov V.; Waheed S.; Walker T.; Walsh U.; Warner R.; Waters R.; Wilkins A.; Williams K.; Wilson G.; Wiltsher M.; Wooler B.; Wright C.; Wright M.; Wyld L.; Youssef M.; Zabkiewicz C.; Zammit C.; Zeidan B.; Zheng D. Short-term safety outcomes of mastectomy and immediate implant-based breast reconstruction with and without mesh (iBRA): a multicentre, prospective cohort study. Lancet Oncol. 2019, 20 (2), 254–266. 10.1016/S1470-2045(18)30781-2. 30639093 PMC6358590

[ref4] Choi J. H.; Gimble J. M.; Lee K.; Marra K. G.; Rubin J. P.; Yoo J. J.; Vunjak-Novakovic G.; Kaplan D. L. Adipose tissue engineering for soft tissue regeneration. Tissue Eng., Part B 2010, 16 (4), 413–426. 10.1089/ten.teb.2009.0544. PMC2946881 20166810

[ref5] Patrick C. W. Breast tissue engineering. Annu. Rev. Biomed. Eng. 2004, 6, 109–130. 10.1146/annurev.bioeng.6.040803.140032. 15255764

[ref6] Chhaya M. P.; Balmayor E. R.; Hutmacher D. W.; Schantz J. T. Transformation of Breast Reconstruction via Additive Biomanufacturing. Sci. Rep. 2016, 6, 28030 10.1038/srep28030. 27301425 PMC4908382

[ref7] Han H. H.; Kim K. K.; Lee K. H.; Kim I. B.; Lee P. K. The Use of a Retromammary Adipofascial Flap in Breast Augmentation for Patients with Thin Soft Tissue. Aesthetic Plast. Surg. 2018, 42 (6), 1447–1456. 10.1007/s00266-018-1215-x. 30132110

[ref8] Noskovicova N.; Schuster R.; Van Putten S.; Ezzo M.; Koehler A.; Boo S.; Coelho N. M.; Griggs D.; Ruminski P.; McCulloch C. A.; Hinz B. Suppression of the fibrotic encapsulation of silicone implants by inhibiting the mechanical activation of pro-fibrotic TGF-β. Nat. Biomed. Eng. 2021, 1 10.1038/s41551-021-00722-z. 34031559

[ref9] Fuoco T.; Ahlinder A.; Jain S.; Mustafa K.; Finne-Wistrand A. Poly(ε-caprolactone-co-p-dioxanone): a Degradable and Printable Copolymer for Pliable 3D Scaffolds Fabrication toward Adipose Tissue Regeneration. Biomacromolecules 2020, 21 (1), 188–198. 10.1021/acs.biomac.9b01126. 31549825

[ref10] Fuoco T.; Finne-Wistrand A. Enhancing the Properties of Poly(ε-caprolactone) by Simple and Effective Random Copolymerization of ε-Caprolactone with p-Dioxanone. Biomacromolecules 2019, 20 (8), 3171–3180. 10.1021/acs.biomac.9b00745. 31268691

[ref11] Liu H.; Ahlinder A.; Yassin M. A.; Finne-Wistrand A.; Gasser T. C. Computational and experimental characterization of 3D-printed PCL structures toward the design of soft biological tissue scaffolds. Mater. Des. 2020, 188, 108488 10.1016/j.matdes.2020.108488.

[ref12] Bao W.; Cao L.; Wei H.; Zhu D.; Zhou G.; Wang J.; Guo S. Effect of 3D printed polycaprolactone scaffold with a bionic structure on the early stage of fat grafting. Mater. Sci. Eng., C 2021, 123, 111973 10.1016/j.msec.2021.111973. 33812601

[ref13] Hutmacher D. W. Quo Vadis Breast Tissue Engineering?. EBioMedicine 2016, 6, 24–25. 10.1016/j.ebiom.2016.03.044. 27211542 PMC4856791

[ref14] Poh P. S. P.; Hege C.; Chhaya M. P.; Balmayor E. R.; Foehr P.; Burgkart R. H.; Schantz J.-T.; Schiller S. M.; Schilling A. F.; Hutmacher D. W. Evaluation of polycaprolactone – poly-D,L-lactide copolymer as biomaterial for breast tissue engineering. Polym. Int. 2017, 66 (1), 77–84. 10.1002/pi.5181.

[ref15] Morrison W. A.; Marre D.; Grinsell D.; Batty A.; Trost N.; O’Connor A. J. Creation of a Large Adipose Tissue Construct in Humans Using a Tissue-engineering Chamber: A Step Forward in the Clinical Application of Soft Tissue Engineering. EBioMedicine 2016, 6, 238–245. 10.1016/j.ebiom.2016.03.032. 27211566 PMC4856786

[ref16] Sommeling C. E.; Van Landuyt K.; Depypere H.; Van den Broecke R.; Monstrey S.; Blondeel P. N.; Morrison W. A.; Stillaert F. B. Composite breast reconstruction: Implant-based breast reconstruction with adjunctive lipofilling. J. Plast. Reconstr. Aesthet. Surg. 2017, 70 (8), 1051–1058. 10.1016/j.bjps.2017.05.019. 28599842

[ref17] Mu X.; Zhang J.; Jiang Y. 3D Printing in Breast Reconstruction: From Bench to Bed. Front. Surg. 2021, 8, 641370 10.3389/fsurg.2021.641370. 34095200 PMC8173201

[ref18] Chhaya M. P.; Melchels F. P. W.; Wiggenhauser P. S.; Schantz J. T.; Hutmacher D. W. Breast Reconstruction Using Biofabrication-Based Tissue Engineering Strategies. In Biofabrication; Forgacs G., Sun W., Eds.; William Andrew Publishing, 2013; pp 183–216.

[ref19] Dalton P. D.; Vaquette C.; Farrugia B. L.; Dargaville T. R.; Brown T. D.; Hutmacher D. W. Electrospinning and additive manufacturing: converging technologies. Biomater. Sci. 2013, 1 (2), 171–185. 10.1039/C2BM00039C. 32481796

[ref20] Shih Y. R.; Chen C. N.; Tsai S. W.; Wang Y. J.; Lee O. K. Growth of mesenchymal stem cells on electrospun type I collagen nanofibers. Stem Cells 2006, 24 (11), 2391–2397. 10.1634/stemcells.2006-0253. 17071856

[ref21] Jiang T.; Carbone E. J.; Lo K. W. H.; Laurencin C. T. Electrospinning of polymer nanofibers for tissue regeneration. Prog. Polym. Sci. 2015, 46, 1–24. 10.1016/j.progpolymsci.2014.12.001.

[ref22] Sill T. J.; Von Recum H. A. Electrospinning: Applications in drug delivery and tissue engineering. Biomaterials 2008, 29 (13), 1989–2006. 10.1016/j.biomaterials.2008.01.011. 18281090

[ref23] Haslauer C. M.; Avery M. R.; Pourdeyhimi B.; Loboa E. G. Translating textiles to tissue engineering: Creation and evaluation of microporous, biocompatible, degradable scaffolds using industry relevant manufacturing approaches and human adipose derived stem cells. J. Biomed. Mater. Res., Part B 2015, 103 (5), 1050–1058. 10.1002/jbm.b.33291. PMC4363300 25229198

[ref24] Mohanty S.; Sanger K.; Heiskanen A.; Trifol J.; Szabo P.; Dufva M.; Emneus J.; Wolff A. Fabrication of scalable tissue engineering scaffolds with dual-pore microarchitecture by combining 3D printing and particle leaching. Mater. Sci. Eng., C 2016, 61, 180–189. 10.1016/j.msec.2015.12.032. 26838839

[ref25] Dalton P. D.; Woodfield T. B. F.; Mironov V.; Groll J. Advances in Hybrid Fabrication toward Hierarchical Tissue Constructs. Adv. Sci. 2020, 7, 1902953 10.1002/advs.201902953. PMC7284200 32537395

[ref26] Hallberg H.; Rafnsdottir S.; Selvaggi G.; Strandell A.; Samuelsson O.; Stadig I.; Svanberg T.; Hansson E.; Lewin R. Benefits and risks with acellular dermal matrix (ADM) and mesh support in immediate breast reconstruction: a systematic review and meta-analysis. J. Plast. Surg. Hand. Surg. 2018, 52 (3), 130–147. 10.1080/2000656X.2017.1419141. 29320921

[ref27] Hansson E.; Edvinsson A. C.; Elander A.; Kölby L.; Hallberg H. First-year complications after immediate breast reconstruction with a biological and a synthetic mesh in the same patient: A randomized controlled study. J. Surg. Oncol. 2021, 123 (1), 80–88. 10.1002/jso.26227. 33051871 PMC7821308

[ref28] Cardoso A.; Santos D.; Martins J.; Coelho G.; Barroso L.; Costa H. Breast ligaments: an anatomical study. Eur. J. Plast. Surg. 2015, 38 (2), 91–96. 10.1007/s00238-014-1024-7.

[ref29] Santos D. C.; Cardoso A.; Martins J. M.; da Luz Barroso M.; Costa H. Suspensory Ligament of the Mammary Gland: A Case Report. Aesthetic Plast. Surg. 2016, 40 (1), 98–101. 10.1007/s00266-015-0589-2. 26695951

[ref30] Rehnke R. D.; Groening R. M.; Van Buskirk E. R.; Clarke J. M. Anatomy of the Superficial Fascia System of the Breast: A Comprehensive Theory of Breast Fascial Anatomy. Plast. Reconstr. Surg. 2018, 142 (5), 1135–1144. 10.1097/PRS.0000000000004948. 30511967 PMC6211786

[ref31] Krouskop T. A.; Wheeler T. M.; Kallel F.; Garra B. S.; Hall T. Elastic moduli of breast and prostate tissues under compression. Ultrason. Imaging 1998, 20 (4), 260–274. 10.1177/016173469802000403. 10197347

[ref32] Samani A.; Zubovits J.; Plewes D. Elastic moduli of normal and pathological human breast tissues: an inversion-technique-based investigation of 169 samples. Phys. Med. Biol. 2007, 52 (6), 1565–1576. 10.1088/0031-9155/52/6/002. 17327649

[ref33] Sommer G.; Eder M.; Kovacs L.; Pathak H.; Bonitz L.; Mueller C.; Regitnig P.; Holzapfel G. A. Multiaxial mechanical properties and constitutive modeling of human adipose tissue: a basis for preoperative simulations in plastic and reconstructive surgery. Acta Biomater. 2013, 9 (11), 9036–9048. 10.1016/j.actbio.2013.06.011. 23811521

[ref34] Fuoco T.; Cuartero M.; Parrilla M.; Garcia-Guzman J. J.; Crespo G. A.; Finne-Wistrand A. Capturing the Real-Time Hydrolytic Degradation of a Library of Biomedical Polymers by Combining Traditional Assessment and Electrochemical Sensors. Biomacromolecules 2021, 22 (2), 949–960. 10.1021/acs.biomac.0c01621. 33502851 PMC7875459

[ref35] Hjort H.; Mathisen T.; Alves A.; Clermont G.; Boutrand J. P. Three-year results from a preclinical implantation study of a long-term resorbable surgical mesh with time-dependent mechanical characteristics. Hernia 2012, 16 (2), 191–197. 10.1007/s10029-011-0885-y. 21972049 PMC3895198

[ref36] Fuoco T.; Almas R. A.; Finne-Wistrand A. Multipurpose Degradable Physical Adhesive Based on Poly(d,l – lactide- co -trimethylene Carbonate). Macromol. Chem. Phys. 2020, 221, 2000034 10.1002/macp.202000034.

[ref37] Jain S.; Yassin M. A.; Fuoco T.; Liu H.; Mohamed-Ahmed S.; Mustafa K.; Finne-Wistrand A. Engineering 3D degradable, pliable scaffolds toward adipose tissue regeneration; optimized printability, simulations and surface modification. J. Tissue Eng. 2020, 11, 1 10.1177/2041731420954316. PMC7498972 32983402

[ref38] Haugh M. G.; Vaughan T. J.; Madl C. M.; Raftery R. M.; McNamara L. M.; O’Brien F. J.; Heilshorn S. C. Investigating the interplay between substrate stiffness and ligand chemistry in directing mesenchymal stem cell differentiation within 3D macro-porous substrates. Biomaterials 2018, 171, 23–33. 10.1016/j.biomaterials.2018.04.026. 29677521 PMC5997298

[ref39] Ghibaudo M.; Saez A.; Trichet L.; Xayaphoummine A.; Browaeys J.; Silberzan P.; Buguin A.; Ladoux B. Traction forces and rigidity sensing regulate cell functions. Soft Matter 2008, 4 (9), 1836–1843. 10.1039/b804103b.

[ref40] Wilkins S. W.; Gureyev T. E.; Gao D.; Pogany A.; Stevenson A. W. Phase-contrast imaging using polychromatic hard X-rays. Nature 1996, 384 (6607), 335–338. 10.1038/384335a0.

[ref41] Paganin D.; Mayo S. C.; Gureyev T. E.; Miller P. R.; Wilkins S. W. Simultaneous phase and amplitude extraction from a single defocused image of a homogeneous object. J. Microsc. 2002, 206 (1), 33–40. 10.1046/j.1365-2818.2002.01010.x. 12000561

[ref42] Feldkamp L. A.; Davis L. C.; Kress J. W. Practical Cone-Beam Algorithm. J. Opt. Soc. Am. A 1984, 1 (6), 612–619. 10.1364/JOSAA.1.000612.

[ref43] Jain S.; Krishna Meka S. R.; Chatterjee K. Curcumin eluting nanofibers augment osteogenesis toward phytochemical based bone tissue engineering. Biomed. Mater. 2016, 11 (5), 055007 10.1088/1748-6041/11/5/055007. 27710925

[ref44] Comley K.; Fleck N. A. A micromechanical model for the Young’s modulus of adipose tissue. Int. J. Solids Struct. 2010, 47 (21), 2982–2990. 10.1016/j.ijsolstr.2010.07.001.

[ref45] Comley K.; Fleck N. The compressive response of porcine adipose tissue from low to high strain rate. Int. J. Impact Eng. 2012, 46, 1–10. 10.1016/j.ijimpeng.2011.12.009.

[ref46] Rouleau L.; Tremblay D.; Cartier R.; Mongrain R.; Leask R. L. Regional variations in canine descending aortic tissue mechanical properties change with formalin fixation. Cardiovasc. Pathol. 2012, 21 (5), 390–397. 10.1016/j.carpath.2011.12.002. 22300500

[ref47] Woodruff M. A.; Hutmacher D. W. The return of a forgotten polymer—Polycaprolactone in the 21st century. Prog. Polym. Sci. 2010, 35 (10), 1217–1256. 10.1016/j.progpolymsci.2010.04.002.

[ref48] Peyton S. R.; Kalcioglu Z. I.; Cohen J. C.; Runkle A. P.; Van Vliet K. J.; Lauffenburger D. A.; Griffith L. G. Marrow-Derived stem cell motility in 3D synthetic scaffold is governed by geometry along with adhesivity and stiffness. Biotechnol. Bioeng. 2011, 108 (5), 1181–1193. 10.1002/bit.23027. 21449030 PMC3357187

[ref49] Pan Z.; Ding J. Poly(lactide- co -glycolide) porous scaffolds for tissue engineering and regenerative medicine. Interface Focus 2012, 2 (3), 366–377. 10.1098/rsfs.2011.0123. 23741612 PMC3363019

[ref50] Meng Z.; He J.; Cai Z.; Wang F.; Zhang J.; Wang L.; Ling R.; Li D. Design and additive manufacturing of flexible polycaprolactone scaffolds with highly-tunable mechanical properties for soft tissue engineering. Mater. Des. 2020, 189, 108508 10.1016/j.matdes.2020.108508.

[ref51] Feng B.; Jinkang Z.; Zhen W.; Jianxi L.; Jiang C.; Jian L.; Guolin M.; Xin D. The effect of pore size on tissue ingrowth and neovascularization in porous bioceramics of controlled architecture in vivo. Biomed. Mater. 2011, 6 (1), 015007 10.1088/1748-6041/6/1/015007. 21206002

[ref52] Li R.; McCarthy A.; Zhang Y. S.; Xie J. Decorating 3D Printed Scaffolds with Electrospun Nanofiber Segments for Tissue Engineering. Adv. Biosyst. 2019, 3 (12), 1900137 10.1002/adbi.201900137. PMC7735424 32648683

[ref53] Dahlin R. L.; Kasper F. K.; Mikos A. G. Polymeric Nanofibers in Tissue Engineering. Tissue Eng., Part B 2011, 17 (5), 349–364. 10.1089/ten.teb.2011.0238. PMC3179616 21699434

