# Supplementary material for: Pliable, Scalable, and Degradable Scaffolds with Varying Spatial Stiffness and Tunable Compressive Modulus Produced by Adopting a Modular Design Strategy at the Macrolevel
Source: ACS Polym Au. 2021 Aug 12;1(2):107–22. doi: 10.1021/acspolymersau.1c00013 (PMC9954393; doi:10.1021/acspolymersau.1c00013)
Supplement: Supplementary file 1 — lg1c00013_si_001.pdf [file lg1c00013_si_001.pdf]

Supplementary Information

Pliable, scalable and degradable scaffolds with varying spatial stiffness and tunable compressive modulus produced by adopting a modular design strategy at the macro level

Authors: Hailong Liu[1,2], Shubham Jain[1,§] , Astrid Ahlinder[1,§], Tiziana Fuoco[1], T. Christian Gasser[2,3,*], Anna Finne-Wistrand[1,*]

[1]Department of Fibre and Polymer Technology, KTH Royal Institute of Technology, Stockholm, Sweden

[2]Solid Mechanics, Department of Engineering Mechanics, KTH Royal Institute of Technology, Stockholm, Sweden

[3]Faculty of Health Sciences, University of Southern Denmark, Odense, Denmark

*Corresponding Author, Email address: annaf@kth.se; gasser@kth.se

[§] Equal contribution is acknowledged between the authors

1. Fabrication of textiles, flat- knitting and non-woven

To assess how the mechanical properties of the multi-layered scaffolds can be tailored depending on the mechanical properties of the textiles, an array of textiles was fabricated from PLA, through flat knitting and die punching. The elastic modulus of the textiles was used in simulation of scaffold according to Table S1.

The flat knitted textiles were made using two yarns, in 1a and 1b tubular stiches only have been used however with an increased yarn tension in 1a. Textile 2 is a non- woven mesh produced through die punching using the same web as have been spun into yarn for the other textiles. By combining textile 1a and 2 to yield textiles 3a-3c, the non-woven can reduce the stiffness of the construct depending on how it is attached to the textile and subsequent treatment of the textile. In 3a the non-woven (2) was placed inside textile 1a, whilst for 3b and c it was attached on the side, 3b was additionally treated with calendaring afterwards. In textile 4 a stitch pattern of 1

rib and 5 tubular was used, then the textile was calendared. For textile 5 the non-woven was attached on the side of textile 4 using die punching and once again calendared. Due to the low mechanical properties and experimental issues related to testing and fabrication the non-woven mesh was only incorporated the simulation only been used in combination with textile 1a and 4 to create textiles 3a-c and 5. Micrographs of the textiles made through different manufacturing techniques and knitting patterns are shown in Figure S1.

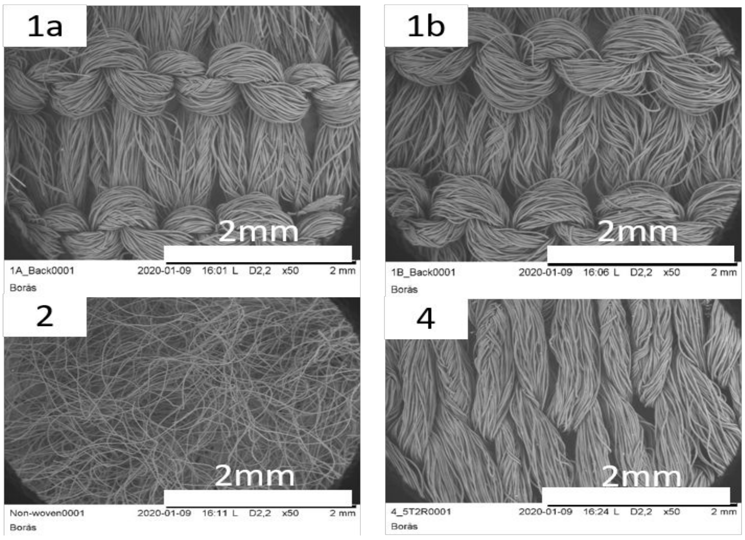

Fig. S1 SEM images of the textiles

Table S1. Description of the textiles used

PLA textiles from Borås		
Type	Tensile modulus, MPa	Description
1a	130	Flat knitted, Flat knitted, tubular stiches only, made with two yarns and high tension afterwards the textile was calendared.<br>High tension, 6,5 * 15 mm
1b	40	Flat knitted, tubular stiches only, made with two yarns and high tension afterwards the textile was calendared.<br>Low tension, 8,5 * 15 mm
2	-	2L = 2 layers (30gr) of web, punched 1 time in the 2 directions, 15 *15 mm
3a	40	Flat knitted made with tubular stiches made of two yarns, the non- woven was placed inside and the whole structure was calendared
3b	20	Flat knitted of tubular stiches using two yarns before calendaring, the non- woven was fixed to the textile

		through die punching before it was once again calendared.
3c	10	Flat knitted of tubular stiches using two yarns before calendaring, the non- woven was fixed to the textile through die punching.
4	60	Flat knitted with the pattern of 1 rib and 5 tubular stitches using two yarns before heat treated with calendaring.
5	30	Flat knitted with the pattern of 1 rib and 5 tubular stitches using two yarns before heat treated with calendaring, non- woven was fixed to the textile through die punching before it was once again calendared.

2. Simulation Cases

The details regarding the simulation of the combined scaffold for compression and tension using 3D printed structures and mesh are further stated in tables S2-S3. The G15 and GS15 structures have previously been analysed and published.[1] The mechanical properties of the textiles in Table S1 were characterized, and the elastic modulus data were used in the simulations below. Information and data from the mesh characterization can be found in the Table S2 and Fig. S2.

Table S2 Simulation case description for the combination design – Compression

Combination design	Scaffold + mesh			
	G15/GS15 Scaffold		Mesh	
Part type	Solid		Solid	
Pore size, mm	0.8-0.8-0.4-0.4-0.8-0.8<br>0.6-0.6-0.3-0.3-0.6-0.6<br>0.4-0.4-0.2-0.2-0.4-0.4		-	
Material	PCL	PCLDX	TIGR® Matrix	PLA textiles from Borås
Elastic modulus, MPa	320	210	40	10, 20, 30, 40, 60, 120
Boundary condition	Displacement equals to 1% strain applied on the rigid plate at top side of the scaffold			
Three designs regarding mesh position relative to the scaffold				

Combination	One side - OS	Two sides - TS	Two sides and middle - TSM
Yellow section - TIGR mesh, Blue section - scaffold			
Model	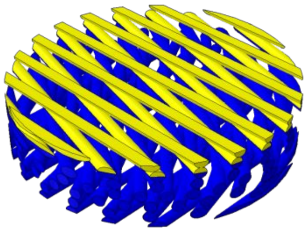	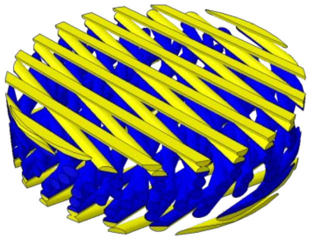	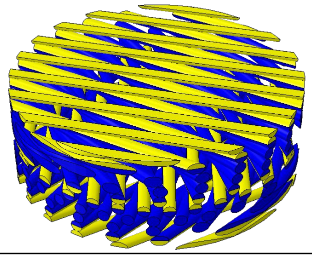
Number of layer - scaffold	6	6	6
Number of layer - mesh	1	2	4
Thickness/ Height, mm	1.95	2.73	3.51
Dimension	Diameter = 10 mm		
Main assumption			
A prefect adhension between the mesh and the scaffold is assumed, i.e. the adhensive surface is plat, smooth, and no penetrations.			

The meshes that are attached to the original G15 scaffold in Table S2 are represented by six textiles/meshes with elastic moduli of 10, 20, 30, 40, 60 and 130 MPa and Poisson's ratio of 0.3. The manufacturing details of these textiles/meshes are given in Table S1, among them, TIGR® Matrix is a commercially available product with an elastic modulus of 40 MPa.

To understand the impact of elastic modulus of the textiles used in Table S2 on the three modular scaffold designs, simulations were run on the three cases OS, TS and TSM, Fig. S2.

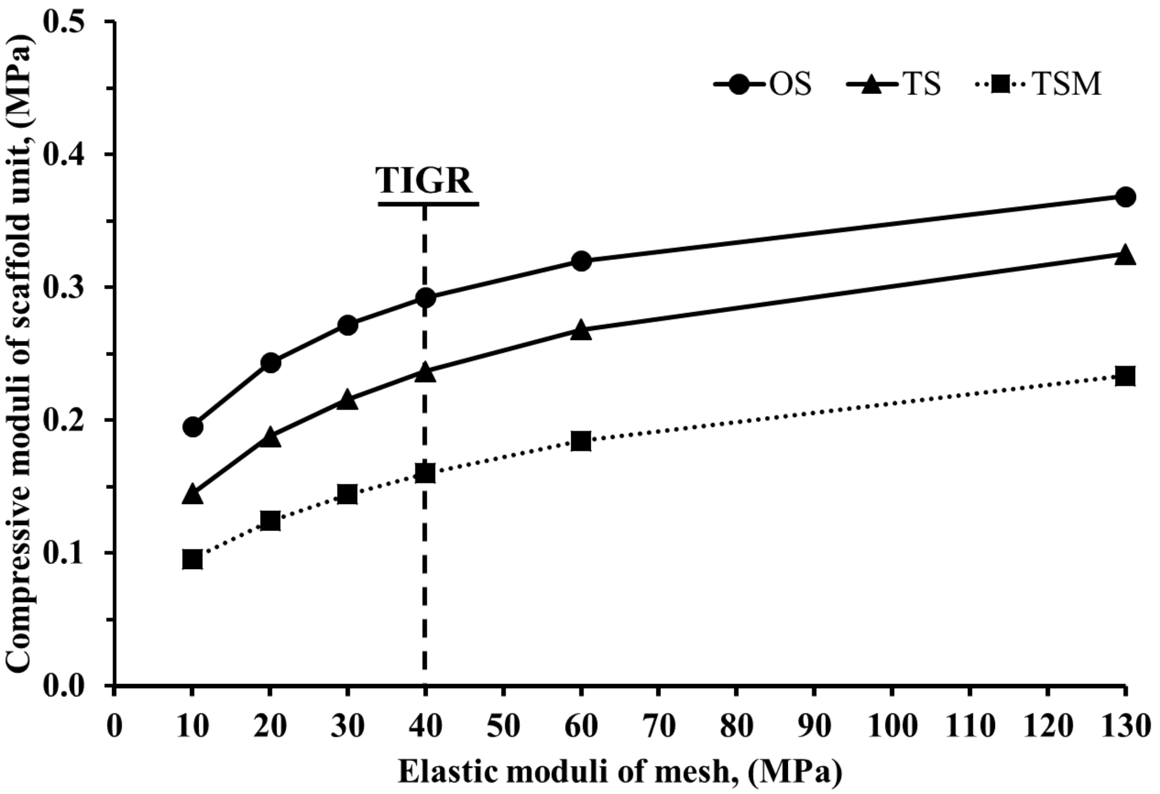

Fig. S2 Simulation of compressive modulus of the modular scaffold designs, OS, TS and TSM depending on the elastic modulus of the textiles

The simulation results in Fig. S2 demonstrate that the computed compressive moduli of the modular scaffold varied as a function of the selected configuration and material properties of the mesh, with OS (0.2 – 0.37 MPa), with TS (0.14 – 0.32 MPa), and with TSM (0.09 – 0.23 MPa) shown in Fig. S2. Due to the commercial availability of the TIGR® Matrix on the market and the midrange values of the simulation with respect to compressive modulus of the modular scaffold design the focus of the manuscript was placed on the use of this textile. More information regarding the influence of mesh type on the compressional and tensional modulus can be seen in Table S3 and S4 as well as Fig. S3 and S4.

Table S3 Simulation case description for the combination design – Tension

Combination design	Scaffold + mesh	
	G15/GS15 Scaffold	Mesh

Part type		Solid	Solid
Pore size, mm		0.8-0.8-0.4-0.4-0.8-0.8<br>0.6-0.6-0.3-0.3-0.6-0.6<br>0.4-0.4-0.2-0.2-0.4-0.4	-
Material		PCLDX	TIGR
Elastic modulus, MPa		210	40
Boundary condition		Displacement equals to 1% strain applied on the rigid plate at left side of the scaffold	
Three designs regarding mesh position relative to the scaffold			
One side - OS		Two sides - TS	Two sides and middle - TSM
Yellow section - TIGR mesh, Blue section - scaffold			
Model	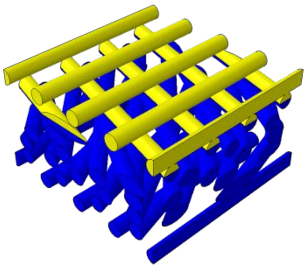	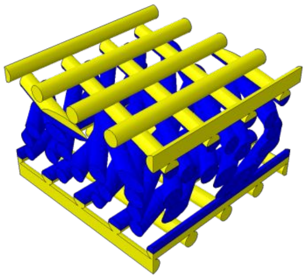	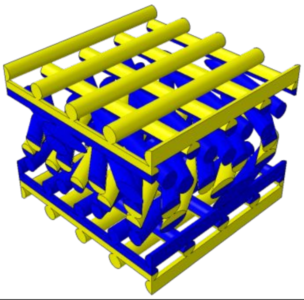
Number of layer - scaffold	6	6	6
Number of layer - mesh	1	2	4
Thickness/Height , mm	1.95	2.73	3.51
Dimension	Length*Width = 4.8 * 4.8 mm		
Main assumption			
A prefect adhension between the mesh and the scaffold is assumed, i.e. the adhensive surface is plat, smooth, and no penetrations.			

The impact of the selection of meshes from Table S1 used in the simulation case in Table S2 on the compressive moduli if G15 or GS15 are printed using PCLDX are shown in Fig. S3. The G15 and GS15 designs are further described in an earlier publication [1].

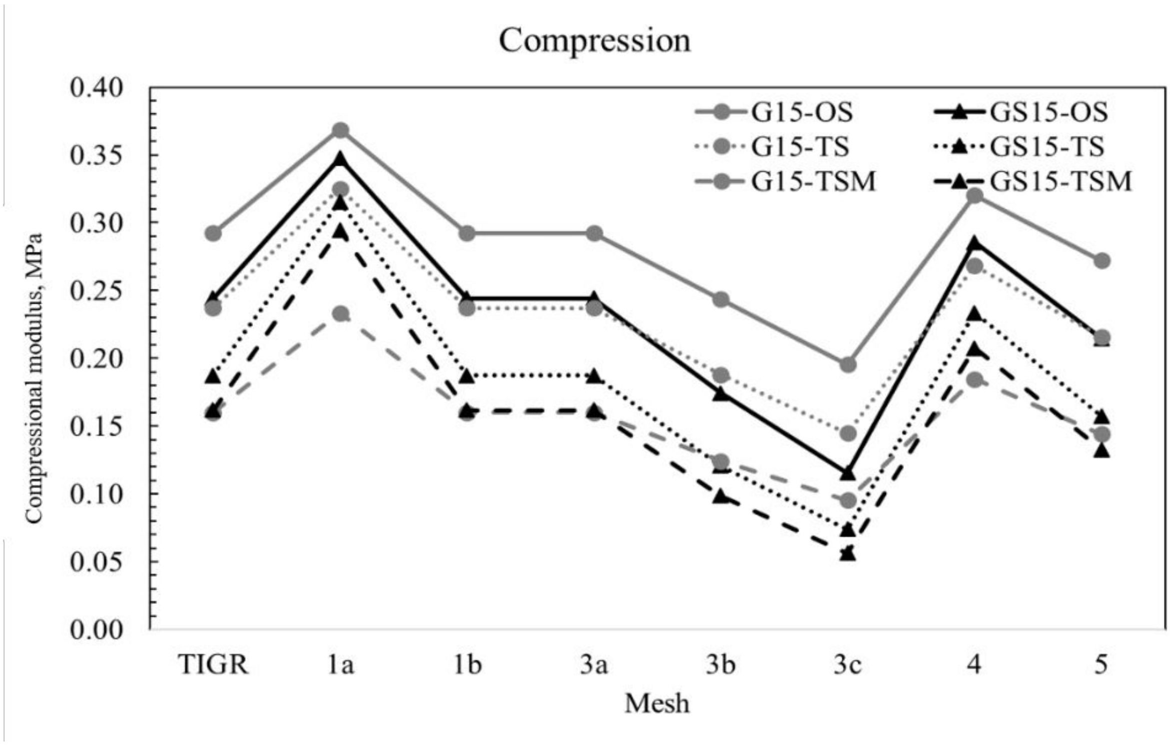

Fig. S3 The influence of mesh type from table S1 on the compressional properties of scaffolds (PCLDX) with different combination designs (the position of mesh to the scaffold) and if the G15 or GS15 were printed.

The impact of the selection of meshes from Table S1 used in the simulation case in Table S3 on the tensional moduli are shown in Fig. S4.

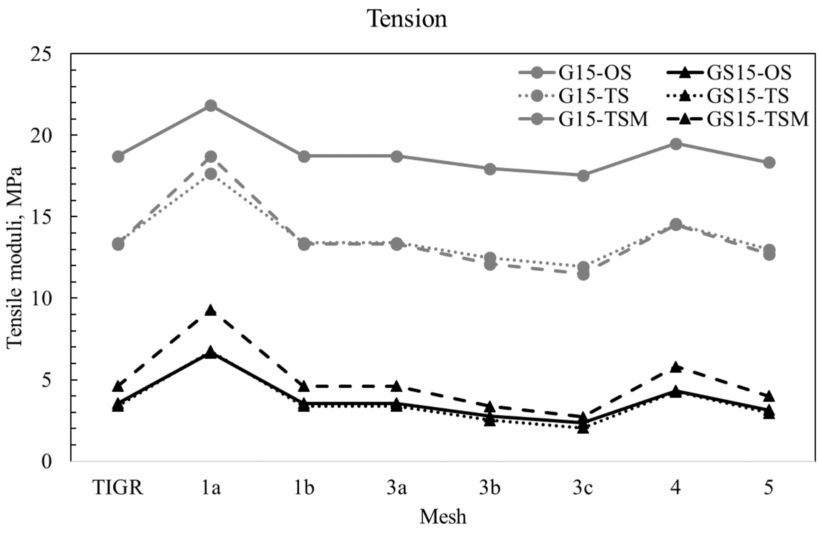

Fig. S4 The influence of mesh type from table S1 on the tensional properties of scaffolds (PCLDX) with different combination designs (the position of mesh to the scaffold) and if the G15 or GS15 were printed.

The simulated case of the effect of the pore size of the printed scaffold to on the tensile moduli from Table S3 is shown in Fig. S5.

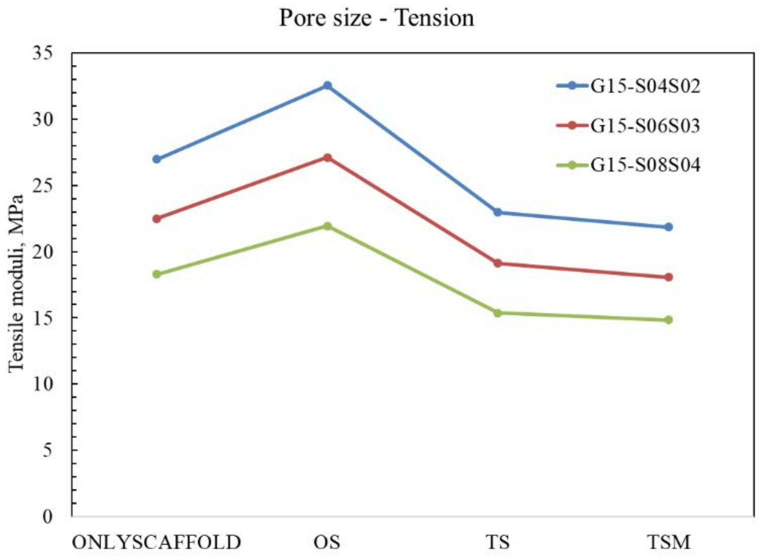

Fig. S5 The effect of the pore size (the strand space between two strands in one layer) on mechanical properties of scaffolds (PCLDX) with different combination designs (the position of mesh to the scaffold) under tension.

To explore the future use of these modular scaffolds for larger devices, the simulation case for a device composted of two scaffolds is shown in Fig. S6.

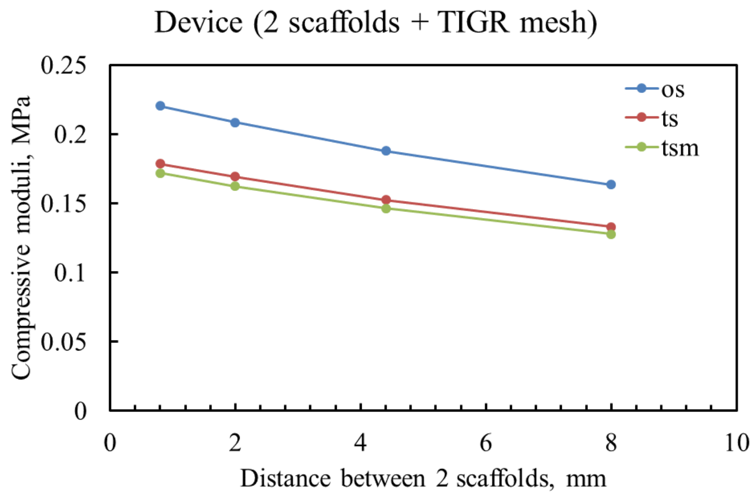

Fig. S6 The compressional moduli of a device that comprises of two scaffolds with the OS, TS and TSM under compression at different distances between them

Due to the isotropic nature of the TSM and G15 scaffold, the values from the x-direction is showed in Fig. S7.

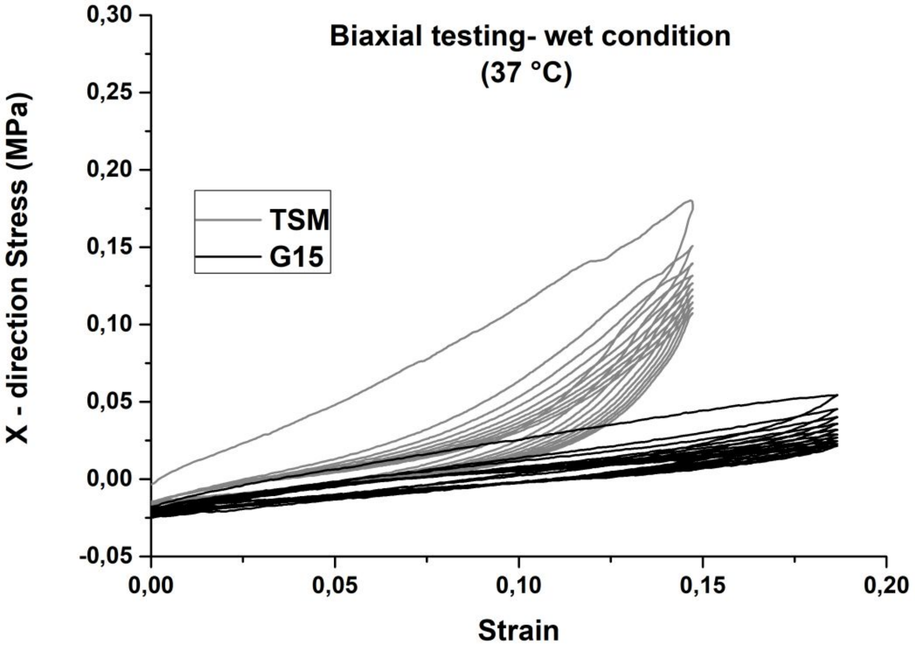

Fig. S7 The stress arising in the x-direction in cyclic testing during 10 cycles of the TSM and G15 scaffold design in wet conditions at 37 °C are shown.

Cell proliferation and attachment at 7 and 11 days of the HDFs are shown with SEM micrographs in Fig. S8

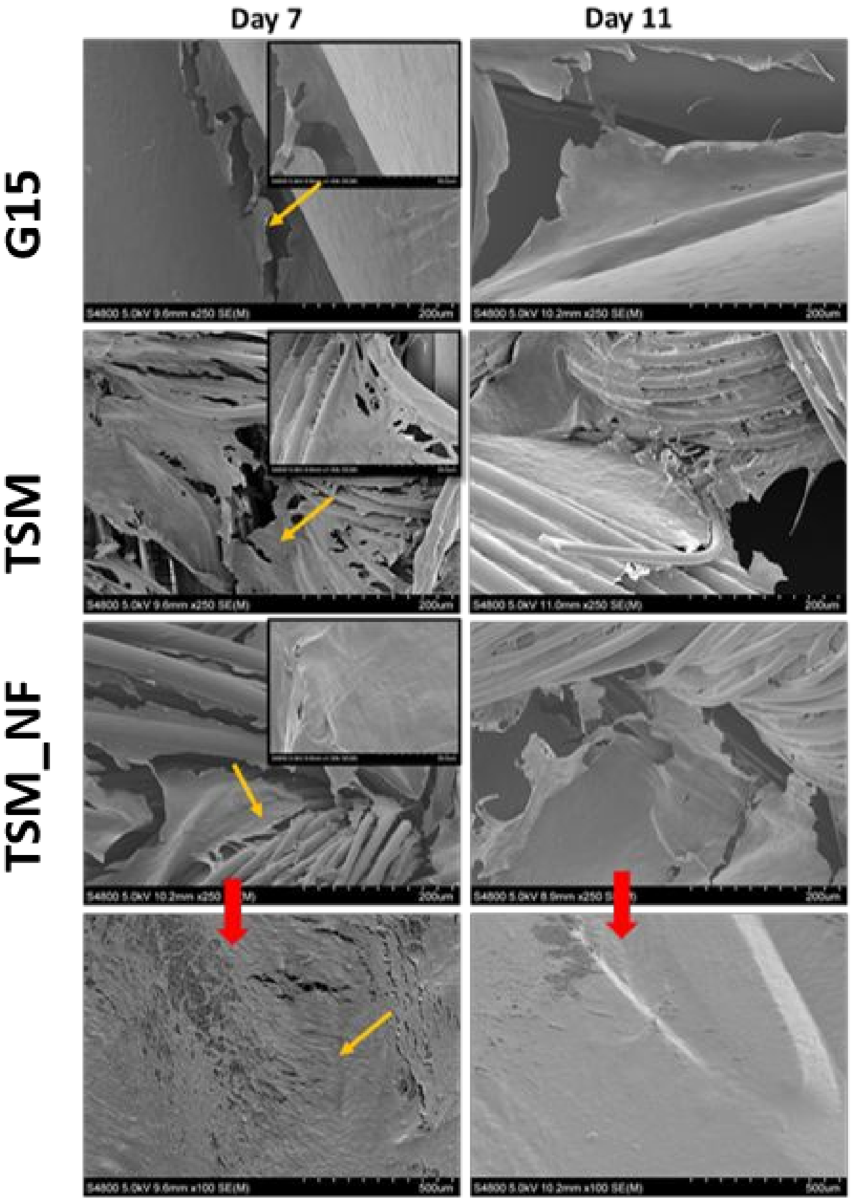

Fig. S8 The proliferation and attachment HDFs in the modular designs are shown at day 7 and day 11 in SEM micrographs, the scale bar showing 200 micrometer. The yellow arrows are indicating the cells onto the scaffold structures. One mesh layer and one 3-layer 3D-printed G15 structure was gently lifted off to expose the nanofiber network. The red arrows show the nanofibrous structure inside the TSM_NF scaffold, with the scale bar showing 500 micrometers.

References

1. Liu, H.; Ahlinder, A.; Yassin, M. A.; Finne-Wistrand, A.; Gasser, T. C., Computational and experimental characterization of 3D-printed PCL structures toward the design of soft biological tissue scaffolds. *Materials & Design* **2020,** *188*, 108488.
